# Biophysical targeting of high‐risk cerebral aneurysms

**DOI:** 10.1002/btm2.10251

**Published:** 2021-09-16

**Authors:** Mark Epshtein, Moran Levi, Afif M. Kraitem, Hikaia Zidan, Robert M. King, Meinrad Gawaz, Matthew J. Gounis, Netanel Korin

**Affiliations:** ^1^ Department of Biomedical Engineering Technion Israel Institute of Technology Technion City, Haifa Israel; ^2^ Department of Radiology, New England Center for Stroke Research University of Massachusetts Medical School Worcester Massachusetts USA; ^3^ Department of Cardiology and Angiology University Hospital Tübingen, Eberhard Karls Universität Tübingen Tübingen Germany

**Keywords:** aneurysm targeting, cerebral aneurysm, glycoprotein VI (GPVI) coating, particle carriers, vascular models

## Abstract

Localized delivery of diagnostic/therapeutic agents to cerebral aneurysms, lesions in brain arteries, may offer a new treatment paradigm. Since aneurysm rupture leading to subarachnoid hemorrhage is a devastating medical emergency with high mortality, the ability to noninvasively diagnose high‐risk aneurysms is of paramount importance. Moreover, treatment of unruptured aneurysms with invasive surgery or minimally invasive neurointerventional surgery poses relatively high risk and there is presently no medical treatment of aneurysms. Here, leveraging the endogenous biophysical properties of brain aneurysms, we develop particulate carriers designed to localize in aneurysm low‐shear flows as well as to adhere to a diseased vessel wall, a known characteristic of high‐risk aneurysms. We first show, in an in vitro model, flow guided targeting to aneurysms using micron‐sized (2 μm) particles, that exhibited enhanced targeting (>7 folds) to the aneurysm cavity while smaller nanoparticles (200 nm) showed no preferable accumulation. We then functionalize the microparticles with glycoprotein VI (GPVI), the main platelet receptor for collagen under low‐medium shear, and study their targeting in an in vitro reconstructed patient‐specific aneurysm that contained a disrupted endothelium at the cavity. Results in this model showed that GPVI microparticles localize at the injured aneurysm an order of magnitude (>9 folds) more than control particles. Finally, effective targeting to aneurysm sites was also demonstrated in an in vivo rabbit aneurysm model with a disrupted endothelium. Altogether, the presented biophysical strategy for targeted delivery may offer new treatment opportunities for cerebral aneurysms.

## 1 INTRODUCTION

Cerebral aneurysms, blood‐filled lesions in brain arteries, occur in about 5% of the population where 0.2% rupture with a mortality rate of nearly 50%.^1^ Currently there are two types of treatment available: endovascular or neurosurgical clipping of the sac.^1^, ^2^, ^3^, ^4^ In other cases, in which the invasive intervention risks may outweigh the benefits, medical management of risk factors coupled with surveillance imaging may be used. Medical management consists mostly of addressing the underlying hypertension,^5^ smoking cessation, and sometimes these treatments are coupled with anti‐inflammatory drugs such as aspirin.^4^, ^6^ The choice of treatment depends on various characteristics of the aneurysm such as size, morphology, and location, as well as patient‐specific factors such as age and clinical status.^7^ However, medical management is not a treatment of the disease, but rather control of perceived risk factors for aneurysm rupture. Medical management is always coupled with surveillance imaging, and any detected change in the aneurysm size, associated with a 12‐fold increase in rupture risk, triggers invasive or minimally invasive treatment.^8^ Notably, continued surveillance imaging and lack of definitive medical treatment creates severe anxiety in patients. Nevertheless, whenever a surgery or endovascular treatment is opted, the risks of significant morbidity or death are high, between 5% and 10%, depending on rupture status or type of devices to be used.^9^, ^10^ However, there is a lack of clear evidence supporting the efficacy of the pharmacological treatment for unruptured aneurysms.^11^ Thus, there is growing interest in developing new methods to diagnose high‐risk aneurysm as well as to allow focal effective drug delivery to aneurysm sites, which can reduce the risk of rupture.

**FIGURE 1:**
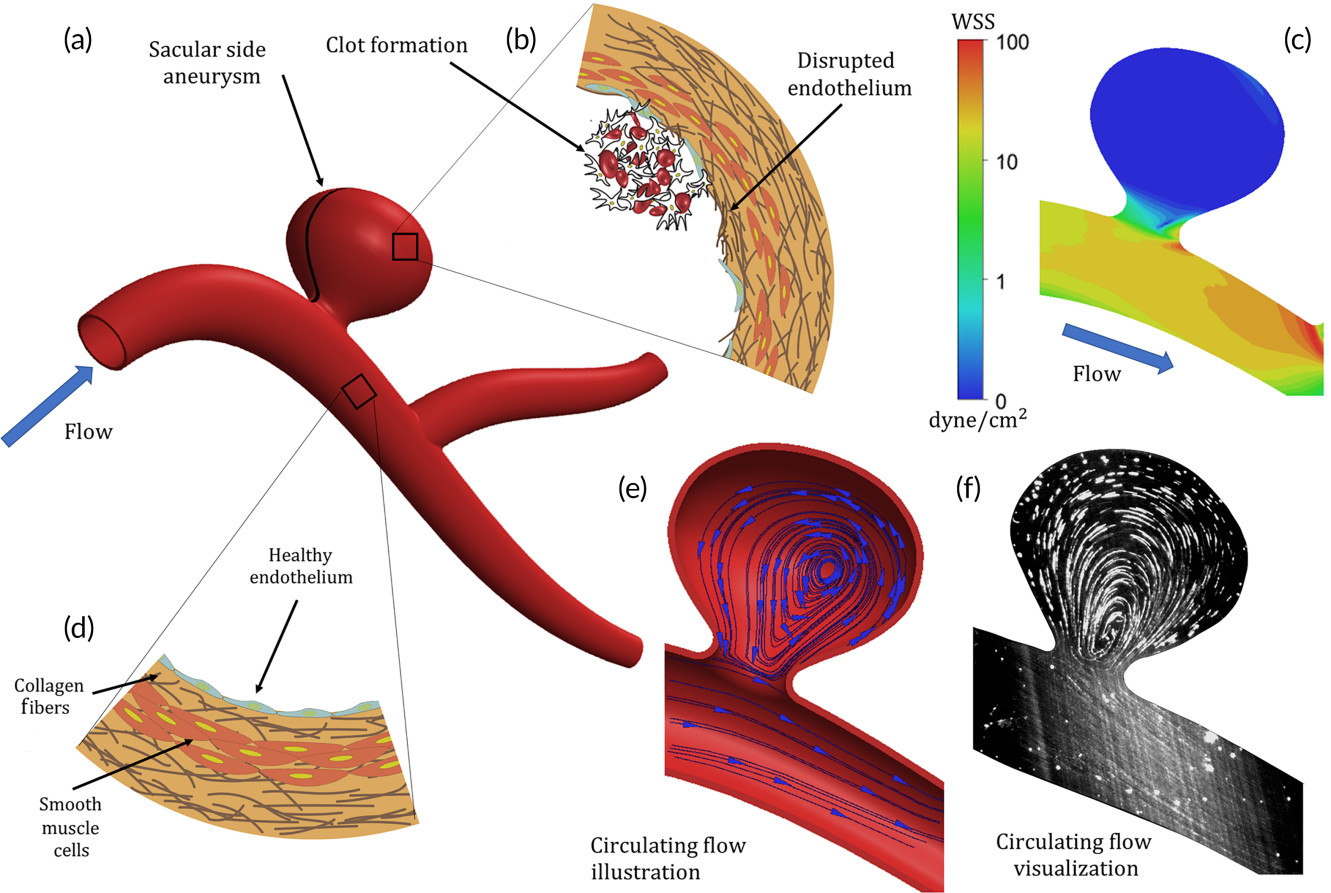
High‐risk cerebral aneurysms are associated with abnormal hemodynamics facilitating pathological biological processes. (a) Illustration of a saccular cerebral side aneurysm. (b) Illustration of disrupted endothelium with collagen fibers protruding through the gaps between the endothelial cells. (c) Computational fluid dynamic (CFD) simulation results showing ultra‐low wall shear stress (<1 dyne/cm^2^) on the surface of the cavity wall. (d) Illustration of healthy endothelium featuring an endothelial cell layer smooth muscle cells and extracellular matrix with collagen fibers. (e) Streamlines inside an aneurysm cavity showing circulatory flow. (f) Light sheet visualization of fluorescent particles circulation inside and aneurysm model

As high‐risk cerebral aneurysms are known to be associated with abnormal physical characteristics (e.g., low‐shear and recirculating flow patterns^1^, ^12^, ^13^) as well as pathological biological processes (e.g., inflammation and thrombosis), these two features and the link between them has been a subject of great research.^14^, ^15^, ^16^ Local biological features associated with high risk of rupture, which include luminal thrombosis and white blood cell infiltration,^1^, ^16^ are tightly related to interactions between a disrupted or activated endothelium and flowing blood cells, either platelet or white blood cells (Figure 1a,b,d). Such interactions occur also in regular inflammation processes and vessel thrombosis scenarios and thus targeted therapeutics have been developed based on utilizing specific biomolecular interactions between platelet/WBC and an inflamed/injured endothelium. Importantly, cerebral aneurysms also possess unique hemodynamics, which play a key role in disease progression. Moreover, risk assessment based on patient‐specific computational fluid dynamic (CFD) simulations has been explored extensively providing evidence that supports the association between pathological hemodynamics and rupture risk.^17^ Pathological hemodynamics, prevalent in aneurysms such low shear recirculating flow (Figure 1c,e,f; also see Supplementary Material [SM] Video S1), are known to activate and disrupt endothelium. Additionally, the pathological hemodynamics in aneurysms result in mass transport and particle dynamics that differ significantly from their characteristics in normal blood vessels, contributing to disease progression. Yet, the unique hemodynamic environment, which drive pathophysiological processes and potentially rupture, has not been explored as a method to enhance localized delivery of drug carriers to sites of aneurysms.

Here, leveraging the endogenous biophysical properties of aneurysms, we develop particulate carriers designed to localize in aneurysm low‐shear recirculating flows as well as to adhere to a disrupted/injured vessel wall, both known characteristics of high‐risk aneurysms. We first show that flow guided targeting to aneurysm cavities can be achieve with micron‐sized, buoyant particles while smaller nanoparticles, widely used in nanomedicines, show no preferable accumulation, as shown in an in vitro aneurysm model subjected to physiological flow. Several targeting ligands may be used to provide biological‐based specificity to the targeting, these include VCAM and ICAM which inflamed endothelium express and have been previously used as targeting moieties.^18^ However, rupture‐prone aneurysms have been shown to correlate with areas of exposed collagen, we thus focused in this study on the use of glycoprotein VI (GPVI) which has been extensively studied and confirmed to target collagen^19^, ^20^ as well as act under low‐medium shear.^21^ We then examined GPVI microparticle targeting in an in vitro reconstructed patient‐specific aneurysm that contained a disrupted/injured endothelium at the aneurysm cavity. Results in this model as well as in an in vivo rabbit aneurysm model with a disrupted endothelium confirmed highly effective targeting to vessel wall disrupted aneurysm sites.

## 2 MATERIALS AND METHODS

### 2.1 Perfusion system

The perfusion system was detailed previously.^22^ Briefly, the system comprises a reservoir with the perfusion media, a peristaltic pump (Watson Marlow C530), an air damper downstream to remove peristaltic pump oscillations, and a linear motor oscillator was connected in parallel (a schematic of the system is shown Figure S3). The pump produced the constant part of the wave form while the linear motor produced the oscillatory part, and the superposition of both produced the desired waveform corresponding to the basilar artery waveform that was obtained from the measurement performed by Zhu et al.^23^ The system could operate in closed circuit as well as in an open circuit mode to clear the unadhered particles at the end of the experiment.

### 2.2 In vitro aneurysm models design and manufacture

The simplified aneurysm models were designed in SolidWorks and 3D printed using a FormLabs clear resin V4 on Form 2 3D printer. The models were based on the same geometry previously used by us^12^, ^24^ and were similar to other models used in the literature.^2^, ^12^, ^25^, ^26^, ^27^ Then, silicone (Elastosil by Wecker) was poured into the mold, left to cure overnight, and then placed in acetone for a few hours to remove the mold. To ensure a hydrophilic surface, the models were then coated with type I collagen using a mixture of 10% collagen (Vitrocol®, Advanced Biomatrix) to phosphate‐buffered saline (PBS).

The patient‐specific aneurysm geometry was the C0074 unruptured aneurysm from the AneuriskWeb depository.^28^ This geometry was chosen due the wide range of shear and flow patterns that were found in CFD simulations. It exhibits shear ranges from less than 0 to 15 dyne/cm^2^, circulatory flow and impingement regions at the neck and the apex.

To obtain a physical model, the aneurysm and part of the parent artery was isolated and fitted in a mold using Solidworks (see SM Figure S1). The mold was then 3D printed from the Formlabs clear resin V4 using a Formlabs Form 2 3D printer. Silicone (Elastosil by Wecker) was poured into the mold, left to cure overnight, and then placed in acetone overnight remove the mold in the same manner as the simplified models.

The patient‐specific models were endothelialized with human umbilical vein endothelial cells (HUVECs by Lonza) on a substrate of mixed collagen (type I human collagen, Vitrocol®, Advanced Biomatrix) and fibronectin (0.1% solution from human plasma, BioReagent). First, the models were filled with a mixture of 10% collagen, and fibronectin 1% NaOH and 89% PBS, and incubated overnight in 5% CO_2_ at 37°C. The second stage is selective endothelialization of the model using HUVECs (Lonza) suspended in media (ECM, ScienceCell) such that exposed collagen was located only in the aneurysm cavity. By trapping an air bubble in the cavity that separated it from the parent vessel with a meniscus formed by surface tension, we have been able to selectively de‐endothelialized the aneurysm cavity without damaging the endothelial cells in the parent artery (see Figure S2 in the SM for details). The resulting models were then fixed using 4% paraformaldehyde solution and stained using 1% green phalloidin antibody staining (Alexa).

### 2.3 Particle preparation

The polystyrene particles used were red and green fluorescent carboxylated 2 μm and green 200 nm microspheres (ThermoFisher). The 2 μm poly(lactic‐co‐glycolic acid) (PLGA) particles were manufactured by the oil‐in‐water emulsion–solvent evaporation method. While PLGA particles are biodegradable, for the timescales examined in this work (several hours), the effect is negligible.^29^ For biophysical targeting, polystyrene particles were coated with GPVI or BSA by conjugating the protein to the carboxyl groups on the particle surface using EDC Sulfo‐NHS chemistry^30^; a summary of particle characteristics appears in Table S1 in the SM as well as detailed protocols published by Levi et al.^31^

### 2.4 Deposition experiments

The fabricated models were connected to the perfusion system and subjected to a pulsatile 200 ml/min flow rate. The system contained 300 ml of 10% (w/v) dextran 40,000 Da (Dextran from *Leuconostoc* spp, Sigma Aldrich) to PBS solution with 3.5 cP viscosity into which particles were added. The solution contained about 300 k 2 μm particles/ml or 300 M particles/ml of the 200 nm particles. The models were perfused with the particles suspension for 60 min in closed circuit followed by a 5‐min open circuit system wash with the same solution but without particles to remove the nonadhere particles from the system. After that, fluorescent images were acquired using an upright Nikon SMZ 2025 microscope.

### 2.5 Analysis and quantification

The analysis of the in vitro results was done by calculating the average fluorescence in the cavity and the parent artery using ImageJ. The obtained values were then divided by the area of the cavity and the parent vessel respectively which were calculated using SolidWorks area properties function of the CAD models. The ratios were then used to calculate the LI using equation 1 (see SM for details).

To quantify the distribution of particles within the cavity, we used a MATLAB® code to calculate the fluorescence in small squares on the side view of the cavity and correlated them to the corresponding squares on a color map of x axis value on the CAD model that was obtained from Ansys CFD post (see SM for details on the process and the MATLAB code).

### 2.6 Computational fluid dynamics

The fluid dynamics simulations were performed in Ansys Fluent® 15.0 R which solved the continuity and momentum equations to obtain the flow field in both the simplified and the patient‐specific models. The simulations were conducted under a constant flow and uniform inlet assumption. The derived flow field was then used to calculate the wall shear stress (WSS) distribution.

The meshing of the models was done using Ansys GAMBIT® resulting in about a million tetrahedral elements for the patient‐specific geometry and a million and the simplified geometry (see SM for more details on simulation conditions and mesh convergence studies).

### 2.7 In vivo

All animal experiments were approved by the University of Massachusetts Institutional Animal Care and Use Committee (AAALAC International). Six New Zealand White rabbits (four female, weight range 2.7–3.6 kg) were used. All procedures were performed under general anesthesia, prior to all surgical procedures, the animals were pre‐anesthetized by a subcuticular injection of glycopyrrolate (0.01 mg/kg) and sustained release buprenorphine for analgesia (0.15 mg/kg). Anesthesia was induced by an intramuscular injection of ketamine (35 mg/kg) and xylazine (5 mg/kg) and maintained with mechanical ventilation of 1%–3% isoflurane. The physiologic status of the animal was assessed using continuous monitoring of respiration rate, heart rate, oxygen saturation level, end‐tidal CO_2_ level, and temperature.

Elastinolysis was performed followed by decellularization of the origin of the right common carotid artery as previously described.^32^ After a minimum of 3 weeks, the animals were returned to the angiosuite and the brachiocephalic trunk selectively catheterized with a microcatheter (SL10, Stryker Neurovascular). The functionalized particles were delivered at a rate of 1 ml/min for a total infusion of 10 ml. After approximately 30 min from the conclusion of nanoparticle infusion, the animals were euthanized by overdose of sodium pentobarbital (150 mg/kg). The aneurysms and control vessels (CVs) were explanted and snap frozen in optimal cutting temperature compound in liquid nitrogen for histological processing. The tissue was then sliced into 45 and 10 μm sequentially. The thicker slices were intended for fluorescent particle counting and were DAPI stained to examine the boundaries of the tissue. The thinner slices were stained using Movat pentachrome to determine both the local histology of the sample and the boundary of the aneurysm lumen (see details in SM).

### 2.8 Statistical analysis

Statistical analysis was done using the paired two sample for means *t*‐test on the LI values with *p* < 0.05 regarded as significant for all statistical tests. The normality of the data was assessed using the Kolmogorov–Smirnov test.

## 3 RESULTS

### 3.1 Natural hemodynamic‐based localization of particles in aneurysm cavities

To study the ability of particles with different physical properties (i.e., size and density) to localize in aneurysm cavities, we performed in vitro experiments in a well‐defined, transparent cerebral side aneurysm models^12^, ^24^ subjected to physiological pulsatile flow while imaging the deposition pattern in the parent vessel and in the aneurysm cavity (see SM for detailed description of the experimental setup). The WSS in our models is low reaching 0 at the apex and averaging around 1.27 dyne/cm^2^ in the cavity while the average in the parent is over 20 dyne/cm^2^.

**FIGURE 2:**
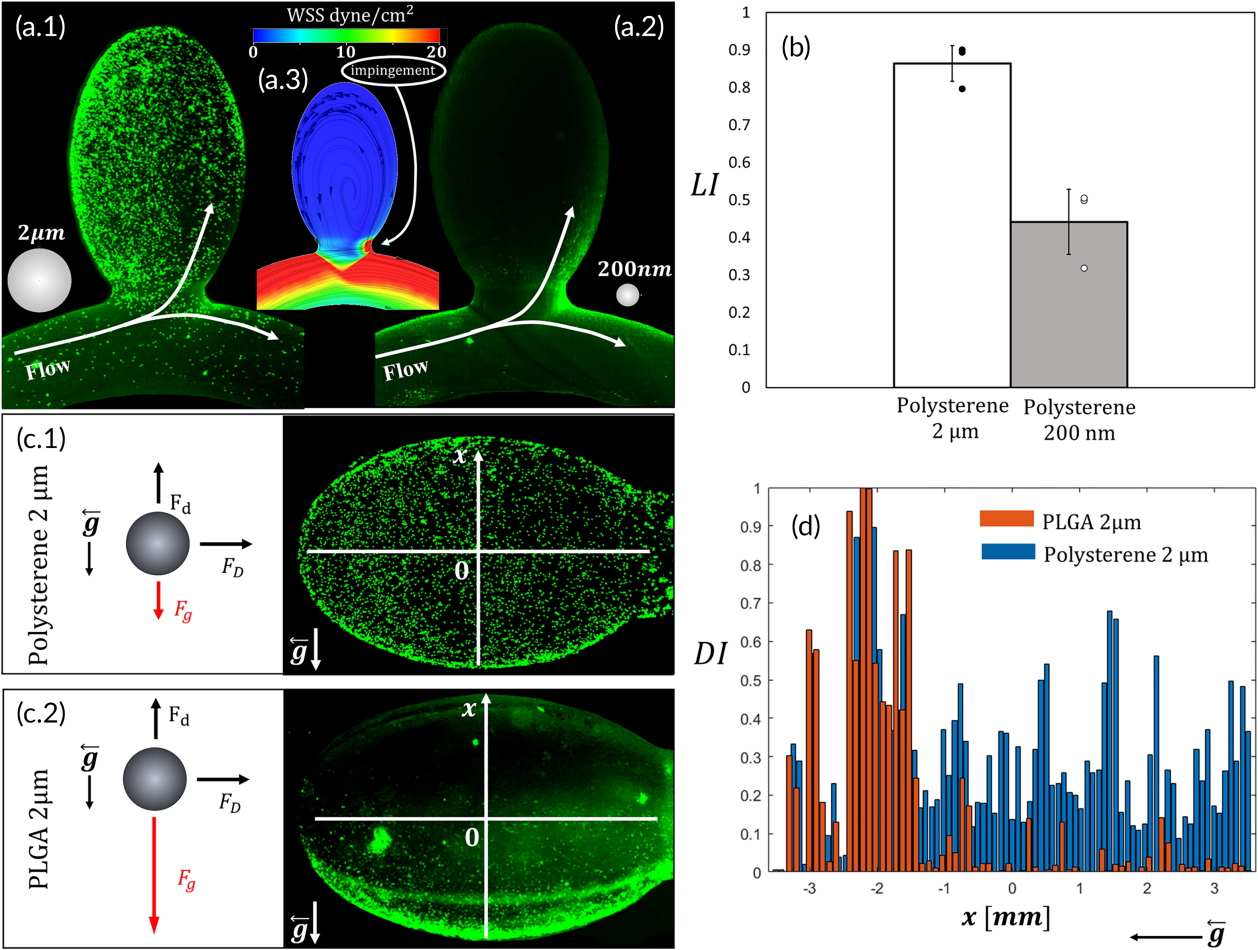
Localization of particles in aneurysm cavities. (a.1) Green fluorescent image of 2 μm carboxylated polystyrene particles in an aneurysm model showing enhanced deposition in cavity associated with low wall shear stress (WSS). (a.2) Green fluorescent image of the 200 nm particles in an aneurysm model showing enhanced deposition in parent artery and the impingement zone associated with high WSS. (a.3) WSS map on the aneurysm geometry showing high shear at the parent artery and low in the aneurysm. The impingement zone is marked by an arrow. (b) Bar graph of the LI for the 2 μm and the 200 nm particles showing that 2 μm had an LI of 0.86 ± 0.05 while 200 nm particles had an LI of 0.44 ± 0.06 (*n* = 3, *p* = 0.004). (a) Side view of the aneurysm cavity showing particles deposition. (a.1) 2 μm PLGA particle have localized predominantly at the bottom side of the cavity according to the direction of gravity. (c.2) 2 μm polystyrene particles coated the entire cavity with no evident bias due to gravity. (d) Deposition intensity (DI) distribution along the x axis showing that the PLGA 2‐μm particles have localized at the bottom of the cavity while the polystyrene particles distributed more evenly

Using our model, we first focused on the effect of particle size by comparing the deposition of 2 μm and 200 nm carboxylated polystyrene spherical particles (CPP). Interestingly, front view deposition images, acquired using fluorescent microscopy, showed completely different patterns for the two particles (Figure 2a). While the 2‐μm CPP mainly localized within the cavity, where low WSS prevails (Figure 2a.1,a.3), the 200‐nm CPP deposited mainly at the distal neck of the aneurysm and the outer curvature of the parent artery (Figure 2a.2), which are associated with high WSS (Figure 2a.3; see also SM Video S2). To quantify the localization of the particles, we defined the localization index (LI) as the ratio of fluorescence concentration (FC) at the cavity (FC_C_) to the sum of FC in the cavity and the parent artery of the aneurysm (FC_C_ + FC_P_), LI = FC_C_/(FC_C_ + FC_P_); thus, this index represents the relative density of fluorescence/particles in the aneurysm compared to the artery (e.g., LI = 0.5 represents no difference between the aneurysm in the parent artery, while LI = 1 represents that all of the adhered particles localized at the aneurysm cavity).

Quantification of the depositions shows that the 2 μm CPP have an LI of 0.86 ± 0.05 which means that the concentration of particles in the aneurysm cavity is 7.4 ± 1.5 times higher than in the parent artery. For the 200 nm CPP, the LI is 0.44 ± 0.06 meaning that the ratio between the aneurysm and the parent artery is only 0.7 ± 0.1; thus, there is actually a slightly higher average concentration of particle deposition in the parent artery. Hence, 200 nm widely used in vascular targeted delivery are less suitable to hemodynamically target aneurysms, while the 2 μm CPPs, naturally target the aneurysm cavity based on its endogenous hemodynamics. While gravity has little effect under normal arterial flow conditions, under the low velocity hemodynamics existing in aneurysms, gravity forces may become dominant and affect the targeted delivery of drug carriers.^24^ These forces depend on the relative acceleration of the particle toward the wall. All the particles in this study have very small relaxation time suggesting that most of them reach terminal velocity before hitting the wall in which case F_d_ = F_g_ = 2.9·10^−18^N (see SM for details on calculation), where F_d_ and F_g_ are the drag and gravity forces, respectively. To test the influence of particle density on deposition under the influence of gravity, side‐view fluorescent images of the aneurysm model were acquired and compared between 2 μm CPPs (*ρ* = 1.05 gr/ml) and 2 μm PLGA particles (*ρ* = 1.3 gr/ml), see Figure 2c. While, the 2‐μm polystyrene particles almost evenly covered both the top and the bottom of the cavity, the 2‐μm PLGA particles, which are 0.15 g/cm^3^ heavier than the fluid, have settled only at the bottom side of the cavity (Figure 2c.2). We then quantified the distribution of the particle deposition in the cavity along the gravity direction with respect to the symmetry axis (*y* = 0) by exploring the normalized fluorescence deposition intensity (DI) as a function of *y*. As shown in Figure 2d, the heavy PLGA particles are almost entirely localized at the negative y values ranging from −3.5 to 0 mm (center point at *y*_c_ = −2.4 ± 0.3 mm) while the polystyrene particles cover the entire range from −3.5 to 3.5 mm with only a slight intensity bias in the direction of gravity (center point at *y*_c_ = −0.23 ± 0.1 mm). The PLGA particles had a similar LI to the polystyrene particles (LI = 0.8 ± 0.1) which is likely due to the favorable gravity configuration in the experiment. However, the PLGA particles settle only on one side of the aneurysm which could be problematic for localized delivery. Moreover, in an unfavorable gravity situation, the PLGA particles will drift away from the aneurysm cavity thus reducing their aneurysm targeting. Thus, in order to properly naturally target the entire aneurysm cavity via flow, buoyant micron sized particles should be utilized.

### 3.2 Targeting endothelium disturbed/injured aneurysms

**FIGURE 3:**
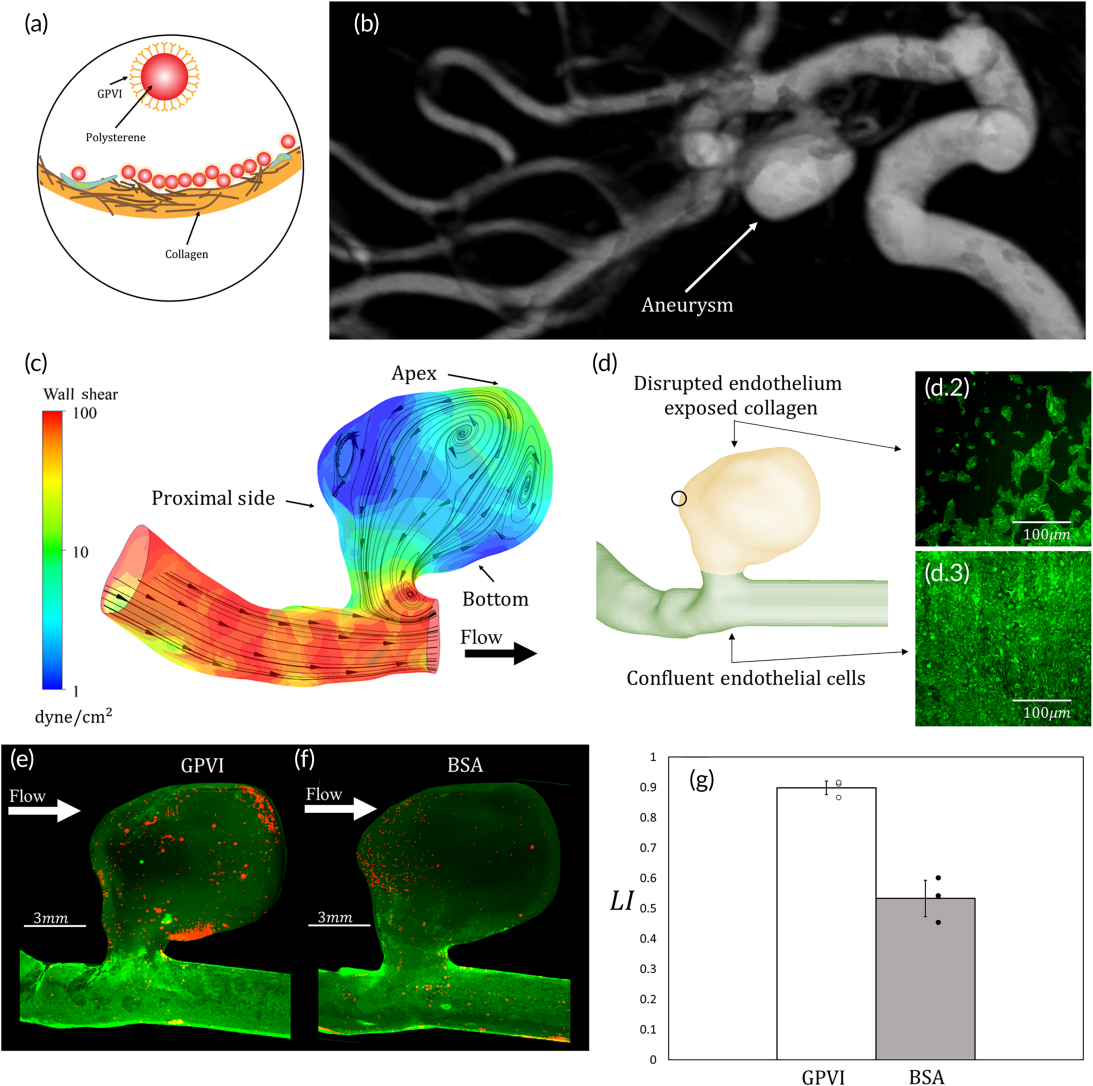
Targeting an endothelium disturbed/injured patient‐specific aneurysm. **(**a**)** Illustration of GPVI functionalized particles adhering to exposed collagen. **(**b**)** Reconstruction of the CT scan of the internal carotid aneurysm obtained from AneuriskWeb repository (scan C0074). (c) Computational fluid dynamic (CFD) results on the patient‐specific geometry showing a low wall shear stress (WSS) recirculating flows within the aneurysm cavity. **(**d**)** Illustration of the injury model with endothelialized parent artery and exposed collagen in the cavity. **(**d.2) Fluorescent microscopy image of green stained ECs forming a disrupted endothelium. (d.3) Fluorescent microscopy image of green stained ECs forming a confluent layer. (e) Fluorescent microscopy image of the aneurysm model where the parent artery is covered with ECs (green) and GPVI‐coated 2‐μm polystyrene particles (red) localizing within the aneurysm cavity. (f) Fluorescent microscopy image of the aneurysm model where the parent artery is covered with ECs (green) and 2 μm polystyrene bovine serum albumin (BSA) particles (red) showing no preference to the cavity. **(**g) Bar graph of the ratio of particle concentrations between the aneurysm cavity and the parent artery. For GPVI‐coated particles, the ratio between the concentration within the aneurysm to the parent artery is 9.1 ± 2.2 while for the BSA‐coated particles the ratio is 1.2 ± 0.4 (*n* = 3, *p* = 0.017)

To add biological specificity to the 2 μm CPP aimed at targeting endothelium disturbed/injured aneurysms, we functionalized particles with the natural main receptor on platelets to collagen, GPVI 4420 ± 1810 molecule/μm^2^, see Figure 3a and details in SM. This 2‐μm GPVI formulation adheres with high affinity to collagen under low WSS (<10 dyne/cm^2^) while has decreased adhesion under higher values of WSS (>10 dyne/cm^2^) known to exist in human arteries, as shown in microfluidic experiments.^22^, ^31^ We then tested the functionality of these particles in a setting of a cerebral side aneurysm, by perfusing the GPVI particles (300 k particles/ml over 60 min) in a patient‐specific reconstructed in vitro aneurysm model (AneuriskWeb repository scan C0074), see Figure 3b MRA scan and corresponding CFD simulation showing the recirculating flow field in Figure 3c. In these models, to recapitulate the proper human physiological environment, first, the model was coated with fibronectin and collagen and then human endothelial cells (HUVECs) were grown to confluence in it covering both the parent artery and the aneurysm cavity. Then, a mechanical injury to the endothelium within the cavity was performed, as detailed in the SM. Thus, while the parent Fartery remained completely endothelialized, the aneurysm cavity presented a disturbed/injured endothelium with exposed collagen, as illustrated in Figure 3d.1–3. Following perfusion with the GPVI particles, fluorescence microscopy images of the aneurysm where particles appear in red and the endothelial cells in green qualitatively demonstrated that the GPVI‐coated particles have densely accumulated in low shear region in the endothelium injured cavity while avoided depositing in the endothelialized parent artery. This is in contradiction to the deposition pattern of control BSA‐coated particles (10,820 ± 1260 molecule/μm^2^) which showed much sparser deposition (see SM Video S3 and Figures S9 and S10 for more detail on particle behavior and targeting characteristics).

Quantitatively, an LI = 0.9 ± 0.02 exists for the GPVI particles indicating a 9.1 ± 2.2 fold higher concentration in the cavity, see Figure 3g. On the other hand, the LI for bovine serum albumin (BSA)‐coated particles is only 0.53 ± 0.07, showing minor to nonsignificant preferable accumulation within the aneurysm cavity. Altogether, we demonstrated that GPVI particles can localize to the disturbed/injured aneurysm an order of magnitude (9.4 ± 4) more than the BSA‐coated particles of the same size and density.

### 3.3 In vivo targeting of injured aneurysms

**FIGURE 4:**
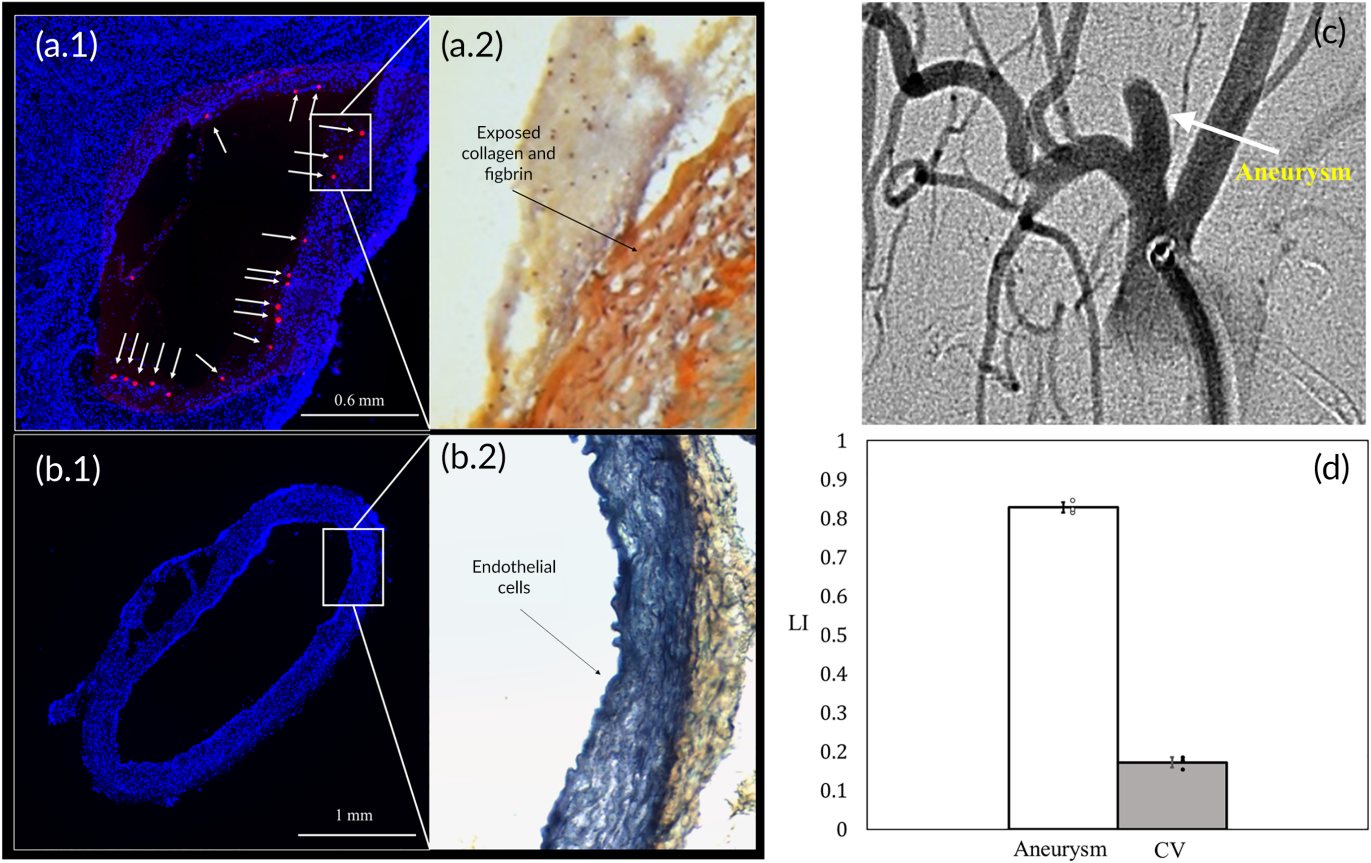
In vivo targeting of injured aneurysms. (a.1) 45‐μm thick DAPI (4′,6‐diamidino‐2‐phenylindole) stained slice taken from a rabbit aneurysm, 18 red fluorescent particles adhered to the inner surface of the aneurysm and marked by white arrows. (a.2) Movats' pentachromic staining of the adjacent slice showing that the aneurysm wall is almost devoid of endothelial cells with exposed collagen and fibrin to which the particle. (b.1) 45‐μm thick DAPI stained slice taken from a control vessel showing no adhered particles on the vessel wall. (b.2) Movats' pentachromic staining of the adjacent slice showing a healthy endothelial layer. (c) CT angiography image of an elastase induced aneurysm in rabbit. **(**d**)** LI distribution between the aneurysm and the control vessel (CV) showing a 4.9 ± 0.6 fold higher accumulation within the cavity (*n* = 3, *p* < 0.0008)

To demonstrate in vivo the ability of the GPVI functionalized polystyrene microparticles to target injured aneurysms, we performed in vivo experiments in elastase induced aneurysm models in rabbits where injury was produced by decellularization during aneurysm creation using SDS,^32^ see details in Methods section. Figure 4a.1 shows a representative, 45‐μm thick section from an aneurysm, that was DAPI (4′,6‐diamidino‐2‐phenylindole) stained for cell nuclei and viewed under a fluorescent microscope revealing multiple red fluorescent particles that adhered to the injured aneurysm wall. Movats' pentachromic staining of the adjusted slices reveals that the injured aneurysm wall is almost devoid of endothelial cells with exposed fibrin and collagen, to which the particles adhere (Figure 4a.2). In contrast, an endothelialized common carotid artery, which served as a distal CV, had almost no adherent particles (Figure 4b.2). Quantitatively, the LI distribution between the aneurysm and the CV, presented in Figure 4d, shows an LI of 0.83 in the aneurysm, which indicate that the particle concentration within the aneurysm is 4.4–5.5 higher than in the CV.

## 4 DISCUSSION

In this work, we have demonstrated an aneurysm‐targeting strategy which leverages the biophysical characteristic of cerebral aneurysms: (1) The endogenous pathological hemodynamics—low WSS, recirculating flow; and (2) the biological environment of high‐risk aneurysm where the endothelium is disrupted and collagen becomes exposed. We have shown that micron‐sized particles (2 μm), and surprisingly not nanoparticles (200 nm), will naturally localize at the aneurysm cavity and that these particles should be neutrally buoyant to minimize the gravitational bias affecting deposition within the aneurysm cavity. Finally, we have demonstrated that the GPVI‐coated microparticles efficiently localize to injured aneurysm and adhere to the wall, as shown in an injured patient reconstructed in vitro aneurysm model and in vivo in an elastase induced rabbit aneurysm model.

The developed strategy is based on a double‐lock mechanism, which combines the biological features of the disease conditions with its biophysical characteristics, and thus increases the specificity of the targeting. Coating of drug carriers with injury or inflammatory ligands^33^ or using white blood cells for carrying drug carriers^34^ would target any inflammation/injury sites in the body and thus would also affect areas in the body where such process plays a key role in normal physiology. Our suggested double‐lock mechanism approach integrating aneurysm biophysics is not only inflammation/injury specific but aneurysm specific based on it flow characteristics. More generally, biophysical targeting approaches which integrate the physics of disease have a wide range of applications in many disease conditions, such as: plaques, tumors and clots, which are known to have unique pathological physical characteristics.

So far, we focused on the ability to target drug carrier to injured cerebral aneurysms; however, future work should utilize the developed approach for the delivery of diagnostic and therapeutic agents. Diagnostics agents to be used may include agents used for functional molecular imaging such as: metal ions such as gadolinium for MRI and manganese‐based paramagnetic contrast agents.^35^, ^36^, ^37^ When administrated systemically, such agents suffer from a low signal‐to‐noise readout and thus focal delivery of such agents may significantly enhance their ability to provide patient‐specific clinical information on risk of rupture. This information is key in deciding whether a surgery or endovascular treatment must be performed. On the other hand, localized delivery of therapeutic agents may open new avenues in nonsurgical pharmacological treatment for unruptured aneurysms delivering potent drugs that can inhibit inflammation^38^ as well as remodeling processes that facilitate aneurysm rupture.^19^, ^39^ Ultimately, combining such targeted therapeutics and diagnostics capabilities can allow to both treat aneurysm noninvasively as well as monitor the efficacy of treatment.

Nevertheless, there are some limitations to the current study including that the particles are relatively large and may accumulate and block small capillaries or be cleared rapidly from circulation and accumulate in the liver or lungs.^40^, ^41^ However, while the particles we have used in this study are not intended for use in a clinical setting, there are several ways to implement such micron or sub‐micron‐sized carriers while minimizing the potential limitations and the filtering effects of relatively large carriers. One of the ways is the use of deformable carriers that mimic the physics of the red blood cells, which has been shown to increase circulation time and reduce accumulation of carriers within the lung and liver as well as reduce patient toxicity.^42^, ^43^ Additionally, RBC‐based drug carriers have recently shown a remarkable improvement in targeting lung and other organs.^44^ Furthermore, such RBC carriers can be made to adhere to exposed collagen,^45^ are about 10 μm in diameter and have relatively long circulation time, and thus could potentially be valuable for targeting high‐risk brain aneurysms.

Another promising strategy is the use of the microparticle via local infusion upstream the site. Infusing the particles close to the target will increase the number of adhering particles on first pass near the target. Such a method was used in our in vivo experiments and has been successfully implemented in delivering nanoparticle using nanoparticle‐loaded RBCs to target inflamed brain vessels.^46^

## 5 CONCLUSIONS

The presented work explored flow‐guided localizations of particles to in vitro cerebral aneurysm models and demonstrated targeting with GPVI functionalized microparticles both in vitro in patient‐specific models and in vivo in rabbit aneurysm models. The presented findings highlight the key role of particle size and density in flow‐guided localization of carriers to aneurysm cavities as well as demonstrate a simultaneous targeting of multiple biophysical properties of the aneurysm. Our results suggest that targeting the biophysical rather than just the biological aspects of a condition is an effective targeting strategy which may be suitable form many other conditions that show significant local physical changes that accompany their biological manifestation.

## CONFLICT OF INTEREST

Meinrad Gawaz is a shareholder of the biotech company AdvanceCor which developed the dimeric soluble GPVI‐Fc (Revacept) molecule. All other authors declared no conflicts of interest.

## AUTHOR CONTRIBUTIONS

**Mark Epshtein:** Conceptualization (lead); investigation (lead); methodology (lead); writing – original draft (lead). **Moran Levi:** Investigation (equal); methodology (equal). **Afif Kraitem:** Investigation (equal); methodology (equal). **Hikaia Zidan:** Methodology (supporting). **Robert King:** Investigation (equal); methodology (equal). **Meinrad Gawaz:** Writing – review and editing (equal). **Matthew Gounis:** Funding acquisition (equal); investigation (equal); supervision (equal); writing – original draft (equal); writing – review and editing (equal). **Netanel Korin:** Conceptualization (lead); investigation (equal); supervision (lead); writing – original draft (equal); writing – review and editing (equal).

### PEER REVIEW

The peer review history for this article is available at https://publons.com/publon/10.1002/btm2.10251.

## Supporting information


**Appendix** S1: Supporting Information
**Figure S1** Fabrication of the physical models
**Figure S2** Injury model fabrication and cell culture
**Figure S3** Perfusion system
**Figure S4** Data acquisition and analysis in the simplified aneurysm models
**Figure S5** Quantification of the gravitational bias in the simplified model
**Figure S6** Data acquisition and analysis in the patient specific aneurysm models
**Figure S7** Data acquisition and analysis in the in‐vivo experiments
**Figure S8** Mesh convergence studies
**Figure S9** In‐vitro patient specific model characteristics
**Table S1** Numerical setup
**Table S2** Summary of particle characteristics

**Video S1** Time lapse movie of 2 μm fluorescent particles circulation inside an in vitro aneurysm model showing slow circulatory flow within the cavity. The field of view was illuminated by a laser sheet (LaVision) and images were taken at a frame rate of 30 frames/s. Flow rate was 200 ml/min. Inset: Picture of a 3D Computer‐Aided‐Design model of the aneurysm and its parent artery.

**Video S2** Time lapse movie showing green fluorescent 2 μm (right) and 200 nm (left) carboxylated polystyrene particles flowing and depositing in a simplified aneurysm model revealing increased deposition of 2 μm particles in the cavity associated with low WSS while the 200 nm deposited more profoundly in the parent artery and the impingement zone associated with high WSS. The final result after a washing step is presented at the end of the video. The images were acquired every 10 second over 50 minutes during the deposition experiments.

**Video S3** A time lapse movie showing red fluorescent 2 μm polystyrene particles in the patient specific aneurysm model revealing the flow patterns and particle trajectories. The particles near the bottom and the proximal side of the cavity are flowing slow enough to observe individual particles which correspond to the ultra‐low shear in the region, while a rapid flow‐direction change is visible near the apex representing higher shear. On the right: image of the particle deposition at the end of the experiment (see Figure 3) The movie shows images acquired every 10 seconds for 50 minutes. Flow rate was 200 ml/min.

**Video S4** A time lapse movie showing a top view of a patient specific aneurysm in vitro model where red fluorescent 2 μm polystyrene coated with BSA deposit only the on the distal neck of a fully endothelialized model. The movie shows images acquired every 10 seconds for 50 minutes. Flow rate was 200 ml/min.

## Data Availability

The data that support the findings of this study are available from the corresponding author upon reasonable request.

## References

[1] Sadasivan C , Fiorella DJ , Woo HH , Lieber BB . Physical factors effecting cerebral aneurysm pathophysiology. Ann Biomed Eng. 2013;41(7):1347‐1365. 10.1007/s10439-013-0800-z 23549899 PMC3679262

[2] Kerl HU , Boll H , Fiebig T , et al. Implantation of pipeline flow‐diverting stents reduces aneurysm inflow without relevantly affecting static intra‐aneurysmal pressure. Neurosurgery. 2014;74(3):321‐334. 10.1227/NEU.0000000000000253 24549048

[3] Chalouhi N , Hoh BL , Hasan D . Review of cerebral aneurysm formation, growth, and rupture. Stroke. 2013;44(12):3613‐3622. 10.1161/STROKEAHA.113.002390 24130141

[4] Brisman JL , Song JK , Newell DW . Cerebral aneurysms. N Engl J Med. 2006;355(9):928‐939. 10.1056/NEJMra052760 16943405

[5] Aoki T , Nozaki K . Preemptive medicine for cerebral aneurysms. Neurol Med Chir (Tokyo). 2016;56(9):552‐568. 10.2176/nmc.st.2016-0063 27053328 PMC5027238

[6] Fisher CL , Demel SL . Nonsteroidal anti‐inflammatory drugs: a potential pharmacological treatment for intracranial aneurysm. Cerebrovasc Dis Extra. 2019;9(1):31‐45. 10.1159/000499077 31039577 PMC7036563

[7] Zhao J , Lin H , Summers R , Yang M , Cousins BG , Tsui J . Current treatment strategies for intracranial aneurysms: an overview. Angiology. 2017;69:17‐30. 10.1177/0003319717700503 28355880 PMC5724574

[8] Villablanca JP , Duckwiler GR , Jahan R , et al. Natural history of asymptomatic unruptured cerebral aneurysms evaluated at CT angiography: growth and rupture incidence and correlation with epidemiologic risk factors. Radiology. 2013;269(1):258‐265. 10.1148/radiol.13121188 23821755

[9] Brinjikji W , Murad MH , Lanzino G , Cloft HJ , Kallmes DF . Endovascular treatment of intracranial aneurysms with flow diverters: a meta‐analysis. Stroke. 2013;44(2):442‐447. 10.1161/STROKEAHA.112.678151 23321438

[10] Health Quality Ontario . Coil embolization for intracranial aneurysms: an evidence‐based analysis. Ont Health Technol Assess Ser. 2006;6(1):1‐114. PMC3379525 23074479

[11] Ajiboye N , Chalouhi N , Starke RM , Zanaty M , Bell R . Unruptured cerebral aneurysms: evaluation and management. Sci World J. 2015;2015:1‐10. 10.1155/2015/954954 PMC4471401 26146657

[12] Epshtein M , Korin N . Mapping the transport kinetics of molecules and particles in idealized intracranial side aneurysms. Sci Rep. 2018;8(1):1‐8. 10.1038/s41598-018-26940-1 29867118 PMC5986792

[13] Bluestein D , Niu L , Schoephoerster RT , Dewanjee MK . Steady flow in an aneurysm model: correlation between fluid dynamics and blood platelet deposition. J Biomech Eng. 1996;118(3):280‐286. 10.1115/1.2796008 8872248

[14] Rayz VL , Boussel L , Ge L , et al. Flow residence time and regions of intraluminal thrombus deposition in intracranial aneurysms. Ann Biomed Eng. 2010;38(10):3058‐3069. 10.1007/s10439-010-0065-8 20499185 PMC2940011

[15] Frösen J , Tulamo R , Paetau A , et al. Saccular intracranial aneurysm: pathology and mechanisms. Acta Neuropathol. 2012;123(6):773‐786. 10.1007/s00401-011-0939-3 22249619

[16] Dooley SA , Hudson JS , Hasan DM . Inflammation in human cerebral aneurysms: pathogenesis, diagnostic imaging, genetics, and therapeutics. Neuroimmunol Neuroinflamm. 2015;2(2):77‐85. 10.4103/2347-8659.154433

[17] Charoenphol P , Huang RB , Eniola‐Adefeso O . Potential role of size and hemodynamics in the efficacy of vascular‐targeted spherical drug carriers. Biomaterials. 2010;31(6):1392‐1402. 10.1016/j.biomaterials.2009.11.007 19954839

[18] Marcos‐Contreras OA , Greineder CF , Kiseleva RY , et al. Selective targeting of nanomedicine to inflamed cerebral vasculature to enhance the blood–brain barrier. Proc Natl Acad Sci USA. 2020;117(7):3405‐3414. 10.1073/PNAS.1912012117/-/DCSUPPLEMENTAL 32005712 PMC7035611

[19] Stellos K , Gawaz M . Platelets and stromal cell‐derived factor‐1 in progenitor cell recruitment. Semin Thromb Hemost. 2007;33(2):159‐164. 10.1055/s-2007-969029 17340464

[20] Nuyttens BP , Thijs T , Deckmyn H , Broos K . Platelet adhesion to collagen. Thromb Res. 2011;127(SUPPL. 2):S26‐S29. 10.1016/S0049-3848(10)70151-1 21193111

[21] Nieswandt B , Watson SP . Platelet‐collagen interaction: is GPVI the central receptor? Blood. 2003;102(2):449‐461. 10.1182/BLOOD-2002-12-3882 12649139

[22] Khoury M , Epshtein M , Zidan H , Zukerman H , Korin N . Mapping deposition of particles in reconstructed models of human arteries. J Control Release. 2020;318:78‐85. 10.1016/j.jconrel.2019.12.004 31812540

[23] Zhu DC , Xenos M , Linninger AA , Penn RD . Dynamics of lateral ventricle and cerebrospinal fluid in normal and hydrocephalic brains. J Magn Reson Imaging. 2006;24(4):756‐770. 10.1002/jmri.20679 16958068

[24] Epshtein M , Korin N . Computational and experimental investigation of particulate matter deposition in cerebral side aneurysms. J R Soc Interface. 2020;17(169):20200510. 10.1098/rsif.2020.0510 32811296 PMC7482554

[25] Sato K , Imai Y , Ishikawa T , Matsuki N , Yamaguchi T . The importance of parent artery geometry in intra‐aneurysmal hemodynamics. Med Eng Phys. 2008;30(6):774‐782. 10.1016/j.medengphy.2007.09.006 18767212

[26] Mulder G , Bogaerds ACB , Rongen P , Vosse FN . On automated analysis of flow patterns in cerebral aneurysms based on vortex identification. J Eng Math. 2009;64(4):391‐401. 10.1007/s10665-009-9270-6

[27] Imai Y , Sato K , Ishikawa T , Comerford A , David T , Yamaguchi T . ATP transport in saccular cerebral aneurysms at arterial bends. Ann Biomed Eng. 2010;38(3):927‐934. 10.1007/s10439-009-9864-1 20012692

[28] AneuriskWeb project website. 2012. http://ecm2.mathcs.emory.edu/aneuriskweb/repository#

[29] Dunne M , Corrigan OI , Ramtoola Z . Influence of particle size and dissolution conditions on the degradation properties of polylactide‐co‐glycolide particles. Biomaterials. 2000;21(16):1659‐1668. 10.1016/S0142-9612(00)00040-5 10905407

[30] Bart J , Tiggelaar R , Yang M , Schlautmann S , Zuilhof H , Gardeniers H . Room‐temperature intermediate layer bonding for microfluidic devices. Lab Chip. 2009;9(24):3481‐3488. 10.1039/b914270c 20024026

[31] Levi M , Epshtein M , Castor T , Gawaz M , Korin N . Glycoprotein VI (GPVI)‐functionalized nanoparticles targeting arterial injury sites under physiological flow. Nanomedicine. 2020;29:102274. 10.1016/j.nano.2020.102274 32712174

[32] King RM , Caroff J , Langan ET , et al. In situ decellularization of a large animal saccular aneurysm model: sustained inflammation and active aneurysm wall remodeling. J Neurointerv Surg. 2021;13:267‐271. 10.1136/neurintsurg-2020-016589 33020207 PMC8632232

[33] Zukerman H , Khoury M , Shammay Y , Sznitman J , Lotan N , Korin N . Targeting functionalized nanoparticles to activated endothelial cells under high wall shear stress. Bioeng Transl Med. 2020;5(2):e10151. 10.1002/btm2.10151 32440559 PMC7237145

[34] Huang B , Abraham WD , Zheng Y , Bustamante López SC , Luo SS , Irvine DJ . Active targeting of chemotherapy to disseminated tumors using nanoparticle‐carrying T cells. Sci Transl Med. 2015;7(291):291ra94. 10.1126/scitranslmed.aaa5447 PMC4687972 26062846

[35] Jasanoff A . MRI contrast agents for functional molecular imaging of brain activity. Curr Opin Neurobiol. 2007;17(5):593‐600. 10.1016/j.conb.2007.11.002 18093824 PMC2883914

[36] Wadghiri YZ , Hoang DM , Leporati A , et al. High‐resolution imaging of myeloperoxidase activity sensors in human cerebrovascular disease. Sci Rep. 2018;8(1):1‐11. 10.1038/s41598-018-25804-y 29769642 PMC5956082

[37] DeLeo MJ , Gounis MJ , Hong B , Ford JC , Wakhloo AK , Bogdanov AA . Carotid artery brain aneurysm model: in vivo molecular enzyme‐specific MR imaging of active inflammation in a pilot study. Radiology. 2009;252(3):696‐703. 10.1148/radiol.2523081426 19546428 PMC2734892

[38] Cheng J , Zhang R , Li C , et al. A targeting nanotherapy for abdominal aortic aneurysms. J Am Coll Cardiol. 2018;72(21):2591‐2605. 10.1016/j.jacc.2018.08.2188 30466517

[39] Hoh BL , Hosaka K , Downes DP , et al. Stromal cell‐derived factor‐1 promoted angiogenesis and inflammatory cell infiltration in aneurysm walls: laboratory investigation. J Neurosurg. 2014;120(1):73‐86. 10.3171/2013.9.JNS122074 24160472 PMC3877706

[40] Decuzzi P , Godin B , Tanaka T , et al. Size and shape effects in the biodistribution of intravascularly injected particles. J Control Release. 2010;141(3):320‐327. 10.1016/j.jconrel.2009.10.014 19874859

[41] Shuvaev VV , Christofidou‐Solomidou M , Scherpereel A , et al. Factors modulating the delivery and effect of enzymatic cargo conjugated with antibodies targeted to the pulmonary endothelium. J Control Release. 2007;118(2):235‐244. 10.1016/j.jconrel.2006.12.025 17270308 PMC1855632

[42] Fish MB , Fromen CA , Lopez‐Cazares G , et al. Exploring deformable particles in vascular‐targeted drug delivery: softer is only sometimes better. Biomaterials. 2017;124:169‐179. 10.1016/J.BIOMATERIALS.2017.02.002 28209527 PMC5341378

[43] Fish MB , Banka AL , Braunreuther M , et al. Deformable microparticles for shuttling nanoparticles to the vascular wall. Sci Adv. 2021;7(17):eabe0143. 10.1126/SCIADV.ABE0143 33883129 PMC8059934

[44] Glassman PM , Villa CH , Ukidve A , et al. Vascular drug delivery using carrier red blood cells: focus on RBC surface loading and pharmacokinetics. Pharmaceutics. 2020;12(5):5‐6. 10.3390/PHARMACEUTICS12050440 PMC7284780 32397513

[45] Samokhin GP , Smirnov MD , Muzykantov VR , Domogatsky SP , Smirnov VN . Red blood cell targeting to collagen‐coated surfaces. FEBS Lett. 1983;154(2):257‐261. 10.1016/0014-5793(83)80160-4 6832367

[46] Marcos‐Contreras OA , Brenner JS , Kiseleva RY , et al. Combining vascular targeting and the local first pass provides 100‐fold higher uptake of ICAM‐1‐targeted vs untargeted nanocarriers in the inflamed brain. J Control Release. 2019;301:54‐61. 10.1016/j.jconrel.2019.03.008 30871995 PMC6510023

