# Supplementary material for: Biophysical targeting of high‐risk cerebral aneurysms
Source: Bioeng Transl Med. 2021 Sep 16;7(1):e10251. doi: 10.1002/btm2.10251 (PMC8780020; doi:10.1002/btm2.10251)
Supplement: Supplementary file 1 — Appendix S1: Supporting Information Figure S1 Fabrication of the physical models Figure S2 Injury model fabrication and cell culture Figure S3 Perfusion system Figure S4 Data acquisition and analysis in the simplified aneurysm models Figure S5 Quantification of the gravitational bias in the simplified model Figure S6 Data acquisition and analysis in the patient specific aneurysm models Figure S7 Data acquisition and analysis in the in‐vivo experiments Figure S8 Mesh convergence studies Figure S9 In‐vitro patient specific model characteristics Table S1 Numerical setup Table S2 Summary of particle characteristics [file BTM2-7-e10251-s002.pdf]

Supplementary Material For:

Biophysical Targeting of High-Risk Cerebral Aneurysms

Mark Epshtein[1], Moran Levi[1], Afif M. Kraitem[2], Hikaia Zidan[1], Robert M. King[2], Meinrad Gawaz[3], Matthew J Gounis[2], Netanel Korin[1]*

[1]Department of Biomedical Engineering, Technion Israel Institute of Technology, Technion City, Haifa 3200003, Israel.

[2]Department of Radiology, New England Center for Stroke Research, University of Massachusetts Medical School, Worcester, MA, United States.

[3]Department of Cardiology and Angiology, University Hospital Tübingen, Eberhard Karls Universität Tübingen, Otfried-Müller Str.10, 72076 Tübingen, Germany

*Corresponding author. Email: korin@bm.technion.ac.il

Supplementary material includes:

Figure S1 Fabrication of the physical models

Figure S2 Injury model fabrication and cell culture

Figure S3 Perfusion system

Figure S4 Data acquisition and analysis in the simplified aneurysm models

Figure S5 Quantification of the gravitational bias in the simplified model

Figure S6 Data acquisition and analysis in the patient specific aneurysm models

Figure S7 Data acquisition and analysis in the in-vivo experiments

Figure S8 Mesh convergence studies

Figure S9 In-vitro patient specific model characteristics

Table S1 Numerical Setup

Table S2 Summary of particle characteristics

Legends for Movies

Model Fabrication

Physical model manufacture

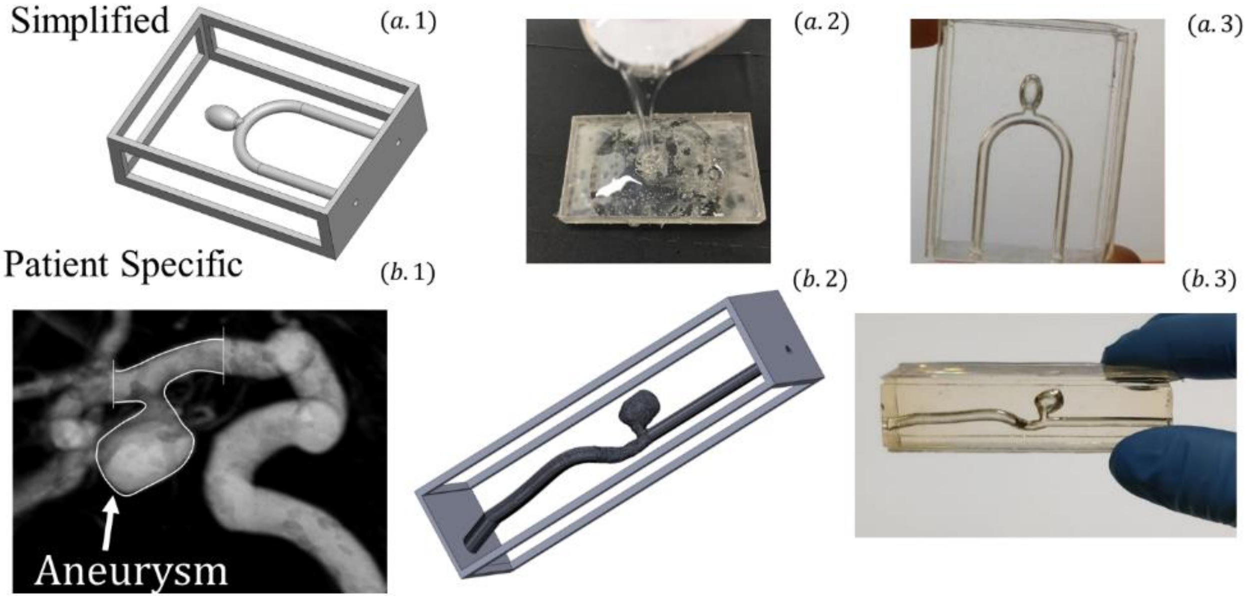

Figure S1 Fabrication of the physical models (a.1) Idealized aneurysm geometry mold - CAD model (a.2) silicone is poured into the mold (a.3) The prepared model after the mold was immersed in acetone overnight. (b.1) MRI scan of a cerebral aneurysm (b.2) CAD of the MRI geometry, processed and fitted into a mold frame. (b.3) The fabricated patient specific model

A CAD model of the mold has been created in SolidWorks (figure S1.a) and 3D printed from a FormLabs clear resin v4 plastic using a Form 2 3D printed. We chose this plastic due to its tendency to breaks up in acetone into soft pieces without melting and sticking to the silicone. Prior to casting the mold were painted with clear lacquer (2X RUST-OLEUM) and left to dry for 24 hours. On the sides of the binding covers (HiClear 200µm) mold were glued to provide a smooth surface on which the side walls to form and a low a perfectly transparent model. The silicone used was Elastosil (by Wecker) which was poured into the mold, was left to cure overnight. Before placing in acetone, the covers were removed, after that the mold was placed in acetone for 24 hours. After the plastic disintegrated, the mold was taken out of the acetone, and the remnants of the plastic removed. Finally, the resulting model was then placed in an oven at 60C to make sure the acetone was completely evaporated (see figure S1.a.3).

The patient specific models were prepared in a similar same manner except that the geometry was obtained from model C0074b available at the AneuriskWeb depository [1]. The geometry was trimmed as shown in figure S1.b.1 and was placed in a mold using SolidWorks (figure S1.b.2), the final prepared model is shown in figure S1.b.3.

Collagen Coating for the simplified models

The simplified aneurysm models, that were not plan to be covered by cells, were coated with type I collagen. The solution contained 9% type I collagen (Vitrocol), 1%V of 0.1M NaOH, and 90% PBS (Dulbecco's) (all percent of volume). The solution was then injected into the models which were then mounted on Intelli centrifuge tube mixer (ELMI RM-2L) and left to incubate for 24 hr rotating on a rotor at 1 RPM in 37°C and 5% CO_2. After 24 hours the models were gently washed with PBS prior to being connected to the perfusion system.

Cell Culture

Prior to seeding the models with cell and models were coated with a mixture of 10% collagen and fibronectin (100 µg/mL, Sigma). The mixture contained 8% type I collagen, 0.9% NaOH and 9% Fibronectin suspended in PBS. The solution was then injected into the models which were then mounted on an Intelli centrifuge tube mixer (ELMI RM-2L) and left to incubate for 24hr rotating in a cell incubator at 1 RPM in 37°C with 5% CO_2. After 24 hours the models were washed with PBS and then seeded with a human umbilical vein endothelial cells (HUVECs, Lonza) cell suspension in endothelial cell medium with fetal bovine serum, endothelial cell growth Supplement and penicillin/streptomycin solution (ECM, ScienceCell, Carlsbad, CA, USA). The cell seeding (5M cells) was done such that the injected solution did not enter the cavity forming a meniscus at the aneurysm neck (figure S2.b). The models were then carefully placed on a rotator and incubated for three days changing medium every 24 hours until about 85% confluency was

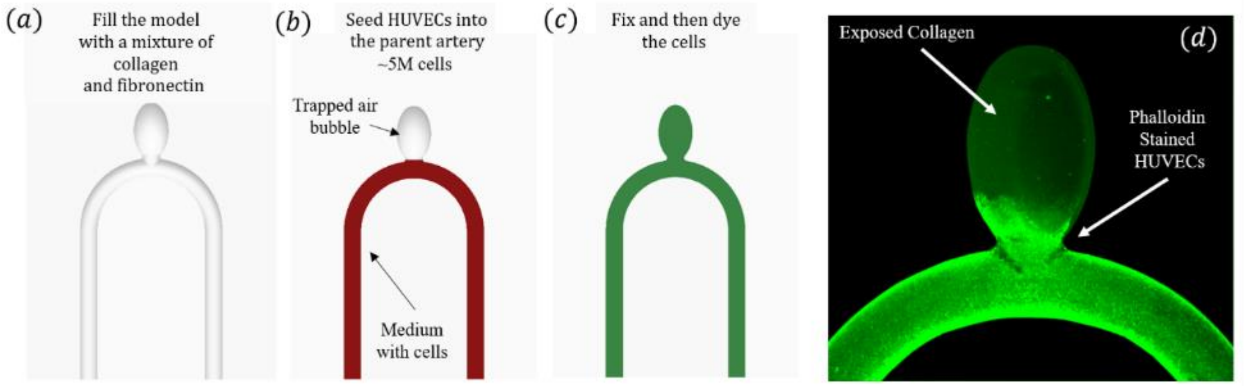

Figure S2 Injury model fabrication and cell culture (a) Infusion of the models with a mixture of 8% type IV collagen, 0.9% NaOH 9%, Fibronectin and 81% PBS (b) Seeding with a human umbilical vein endothelial cell suspension in endothelial cell medium containing around 5M cells. The seeding was done such that the injected solution did not enter the cavity forming a meniscus at the aneurysm neck (c) Fixing the cells with a 4% paraformaldehyde solution for 15 minutes and antibody-staining with Alexa Flour green phalloidin (d) The final model presenting an endothelial layer in parent artery shown in fluorescent green.

reached in the parent vessel, all while maintaining the trapped bubble in the cavity. The models were then washed gently with PBs and exposed to a 4% paraformaldehyde solution for 15 minutes to fix the cells. Finally, the cells were antibody-stained using Alexa Flour green phalloidin (1 mg/ml, 1:20 in PBS) for about 1 hour. The prepared model was then washed gently with PBS before connecting to the perfusion system.

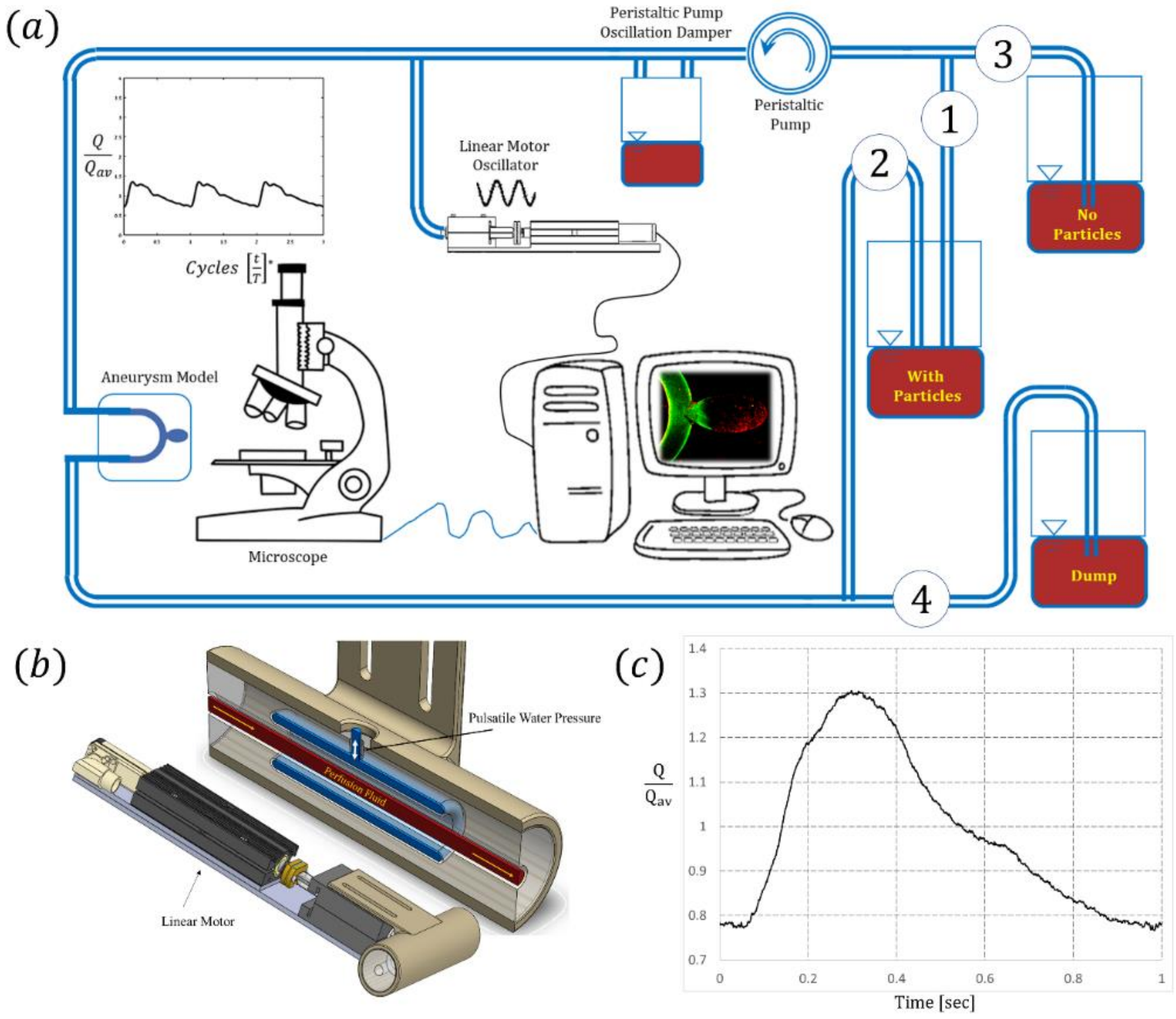

Figure S3 Perfusion system (a) Schematic of the perfusion system. The peristaltic pump draws fluid from a reservoir and infuses it into the damper where the air pressure removes the peristaltic oscillations and produces a smooth continuous flow. To produce the pulsatile waveform a linear motor oscillator pushes periodically a syringe according to a preprogramed motion. The system has two overplayed circuits. When valves 2 and 3 are open and 1 and 4 clos the system operates in closed circuit mode. When valves 2 and 3 are closed and 1 and 4 are open the system operates in open circuit mode. The perfused model is placed under a fluorescent microscope. **(b)** Linear motor oscillator which includes a LinMot® P01-23 linear motor connected to a syringe in a syringe housing. The syringe is connected to a pulsator assembly. The pulsator assembly is comprised of a tube surrounded by a volume of water (blue) when fluid flows through the tube (red) the syringe injects and withdraws volume producing volume changes in the fluid in the tube superposing oscillatory waveform on the constant flow**. (c)** The obtained waveform as was measured with an BP transducer (AD instruments)

The Perfusion System

A perfusion system was developed to emulate the artery's flow waveform in both open and closed-circuit configuration. The system was comprised of a reservoir with the perfusion fluid, a peristaltic pump (Watson Marlow C530) connected to an air damper downstream to remove peristaltic pump oscillations. The pump produces the constant part of the wave form while the linear motor produces the oscillatory part, the superposition of both produces the desired waveform marked in figure S3.a. When valves 3 and 4 are closed and 1 and 2 are open, the system operates in a closed-circuit mode, when valves 1 and 2 are closes and 3 and 4 are open, the system operates in open circuit mode. The actual obtained waveform was measured using a disposable blood pressure transducer (AD instruments) which was amplified by a CW bio- amplifier (figure S3.c).

The flow oscillator which produced the oscillatory part of the flow was made of a linear motor (LinMot® P01-23) connected to a syringe (3 ml lure-lock syringe BD). However, to avoid any leaks, the syringe produced oscillations indirectly through a pulsator assembly which was comprised of a tube surrounded by a volume of water. Thus, when fluid flows through the central tube, the syringe injects and withdraws volume producing volume changes in the fluid in the tube corresponding to the oscillatory part of the flow (figure S3.b). The linear motor was programmed to achieve the desired motion of the linear motor, in a manner identical to the one we used in our previous work and as described there in detail [2].

Perfusion fluid

In all the in-vitro experiments the fluid that was used was a 10% (by weight) dextran 40,000Da (Dextran from Leuconostoc spp, Sigma Aldrich) PBS solution with a 3.5 cP viscosity similar to blood. The solution was prepared by mixing dextran 40 powder in PBS. The viscosity of the mixture was then measured by a CANON FENSKE 50 viscometer.

In-vitro Analysis

Localization Index in the simplified models

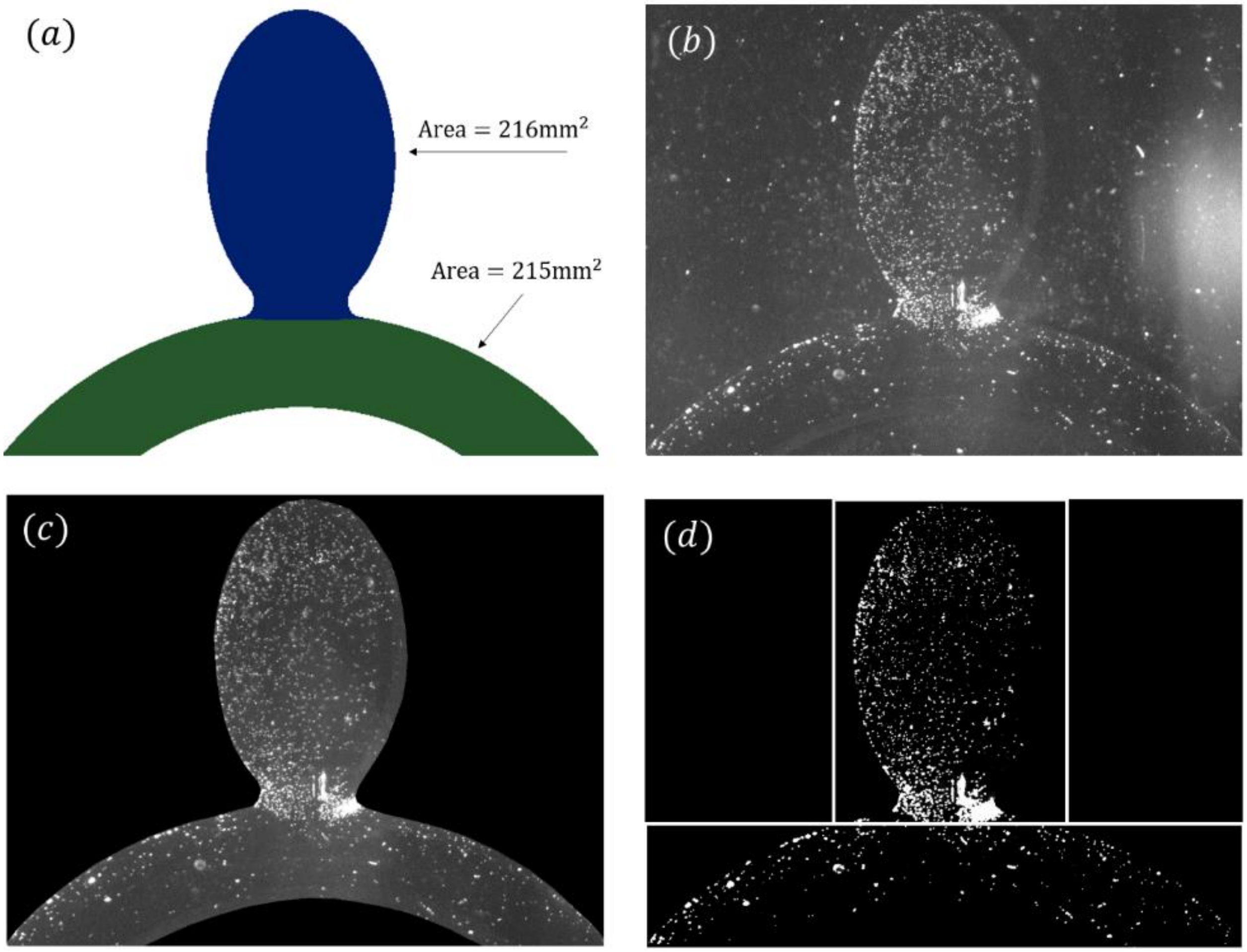

Figure S4 Data acquisition and analysis in the simplified aneurysm models (a) Illustration of the division of the simplified model into two parts with similar surface areas where the aneurysm cavity is in blue and the parent artery in green (b) A fluorescent image of the simplified models after prefusion with carboxylated $2\mu m$ polysterene particles (c) A flourescence image of the simplified model after prefusion with carboxilated $2\mu m$ polysterene particles with the outer backround removed using ImageJ (d) A flourescence image of the simplified models after prefusion with carboxylated $2\mu m$ polysterene particles with the intensity treshold substraccted to show only the particles using ImageJ. Two rectangels separate the model into parts equal to the area division in figure S4.a where the total intesity was calculated using the summation of the non-zero pixels in ImageJ which was then used to calculate FC and LI.

After the simplified models have been perfused for an hour with the particle suspension and then we washed off the un-adhered particles. The models were imaged under a fluorescent microscope to obtained the images shown in figure S4.b. To obtain the average particle concentration in the aneurysm cavity and the parent artery, the model was dividing into two areas with similar surface areas as shown in figure S4.a. Then, the background of the fluorescent images was removed using ImageJ in two stages. First, the surrounding back round was removed (figure S4.c). Second, the

threshold of the image was contacted to the fluorescence of the particles only within the models (figure S4.d). Next, we calculated the sum of the non-zero pixels in the rectangular region marked in figure S4.d and divided them by their corresponding areas to obtain the fluorescence concentration (FC). The localization index (LI) was then calculated by dividing the FC of the aneurysm to the sum of the FC of aneurysm and the parent vessel. This metric allowed us to compare the results of both micro to nano particles despite our inability to image individual nano-particles. Thus, for example, if the LI is 0.5 then particle shows no clear preference of the aneurysm over the parent artery.

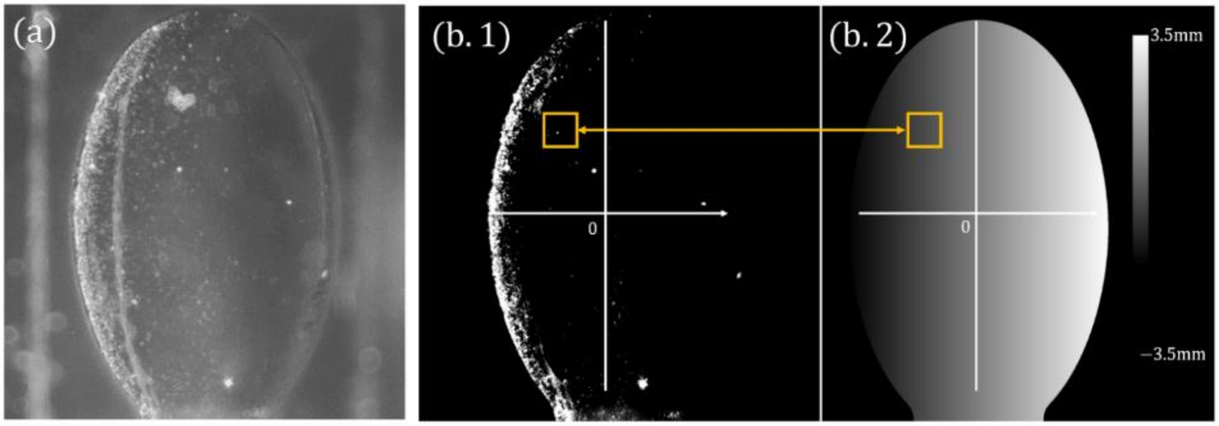

Figure S5 Quantification of the gravitational bias in the simplified model (a) Side view of a simplified aneurysm model after prefusion with $2\mu m$ PLGA particles showing the particles have shifted in the direction of gravity (b.1) Side view of the simplified aneurysm model with the background removed and the intensity threshold subtracted to reveal particles only. The image also shows a superposed axis and the correlation square were the average intensity is measured and correlated to the average x value of the squares position (b.2) A colormap of the y value on the surface of the simplified model from the side view.

Quantification of the gravitational bias in the simplified model

To quantify the distribution of particles on the side view of the simplified aneurysm cavity we have correlated the average fluorescence in a square to the average y value on the corresponding square on the y valued distribution map (figure S5.b). To do this we first removed the background and subtracted the fluorescence threshold to the particles removing autofluorescence from the side view of the physical model (figure S5.b.1). We then plotted the value distribution on the CAD model using Ansys Fluent. To correlate the values, we used a custom written MATLAB® code (see code below) that calculated the average value in a square, loaded it into two vectors. We then plotted one value vs another in a bar graph in EXCEL.

Data Analysis of the Patient Specific model

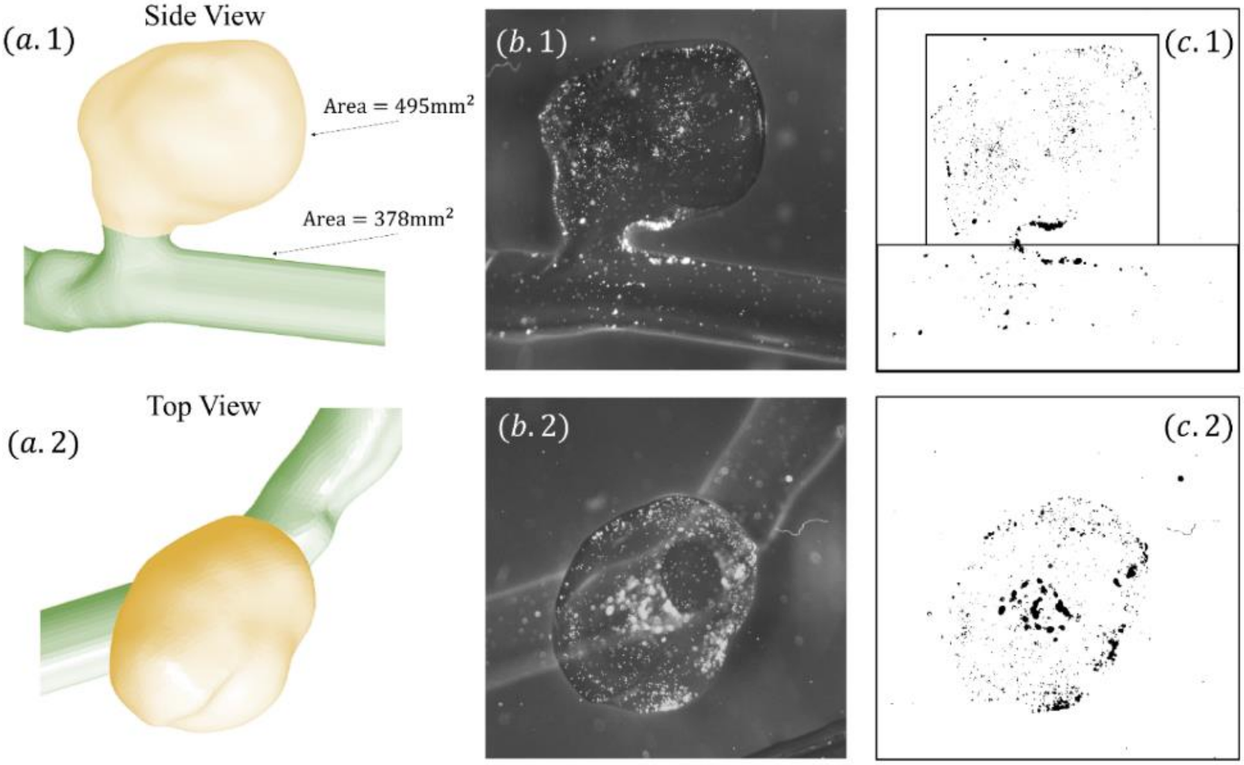

Figure S6 Data acquisition and analysis in the patient specific aneurysm models (a.1) Illustration of the two area divided between the aneurysm cavity (yellow) and the parent artery (green) from the side view of the patient specific model (a.2) Top view of the patient specific model showing the aneurysm in yellow and the parent artery in green (b.1) Side view fluorescent image of GPVI coated polystyrene particles in the patient specific injury model (cells not shown) after perfusion (b.2) Top view fluorescent image of GPVI coated polystyrene particles in a patient specific model following perfusion (cells not shown) (c.1) Side view of the particle fluorescent image after the background was removed and the image turned binary to calculate particles in the demarked squares that correspond to the divided areas (c.2) Top view of the particle fluorescent image after the background was removed

Unlike in the simplified models where we calculated fluorescence, in the patient specific models we counted particles directly on two sides. We achieved this by first removing the background of the fluorescent images and turning them into binary images. Using ImageJ, we estimated the number of particles by counting the number of the detected areas on the side and the top view of the fluorescent images (figures S6.c.1 and S6.c.2). On the side of the aneurysm we divided the image into two rectangles corresponding to the aneurysm and the parent artery (figure S6.c.1) where we calculated the number of particles while on the top view, we calculate only the in one rectangle (figure S6.c.2). We then calculated the average particle concentration (PC) by dividing the sum of particles in the aneurysm on both sides by the area of the aneurysm as calculated using SolidWorks. The localization index was then calculated by dividing the PC in the aneurysm by the sum of PC in the parent artery and PC in the aneurysm.

In vivo

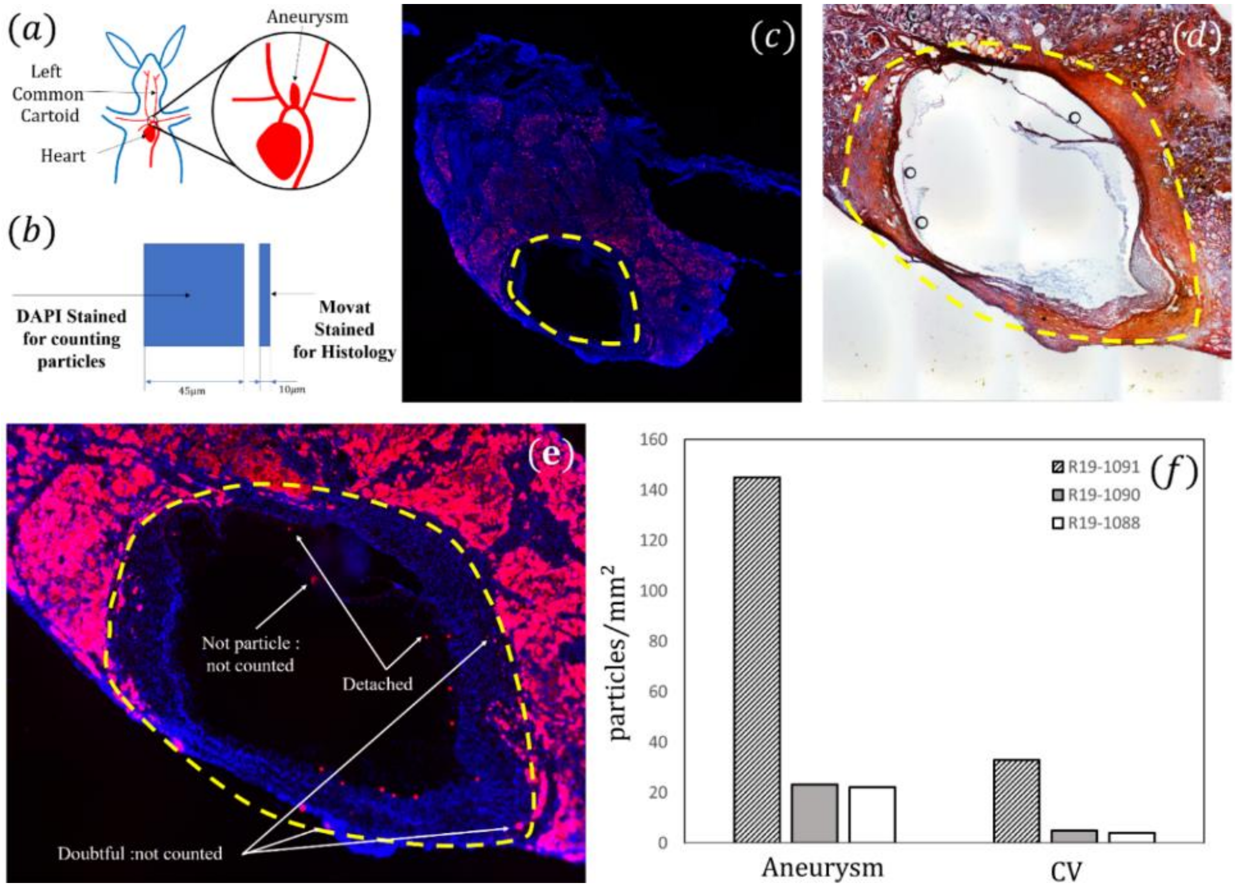

Figure S7 Data acquisition and analysis in the *in-vivo* experiments (a) Illustration of the rabbit vasculature and the location of the aneurysm and the control vessel the left common carotid artery. (b) illustration of the sample cutting procedure: the samples are cut into 45μm and 10 μm sequentially (c) DAPI stained thick slice showing red fluorescent particles as tissue fluorescence as well the aneurysm cavity noted by the yellow dashed line. (d) Movat stained thin slice on with the aneurysm lumen is noted by the dashed yellow line. (e) A DAPI stained thick slice in which the red color was enhance using ImageJ. The image also shows the marked area where particles have been counted and examples of suspected particles that have been rejected in the counting. (f) results of three rabbits R19-1091, R19-1090 and R19-1088 showing similar results for R19-1090 and R19-1088 but an overall increased deposition for the R19-1091. The ratio between the aneurysm and the CV remained the same for all three

Sample Extraction and Preparation

The aneurysm and the control vessel were excised at the end of the experiment and cut into 45μm and 10 μm sequentially as shown in figure S7.b (i.e. and 45 μm slice immediately flowed by a 10 μm slice). The thicker slices were intended for fluorescent particle counting and were DAPI stained to examine the boundaries of the tissue (figure S7.c). The thinner slices were stained with Movat’s pentachromic stain to determine both the local histology of the sample and determine the boundary of the aneurysm lumen (figure S7.d).

I*n Vivo* Analysis

The fluorescent samples (figure S7.c) were each compared to the corresponding Movat stained sample (figure S7.d) to determine the location and boundaries of the aneurysm or the control vessel (CV) inner wall within which we would count the particles. Once the boundary was defined the fluorescent image was loaded into ImageJ and deconstructed into its base colors (RGB). We then enhanced the red image to highlight the particles (figure S7.e). We counted the particles according to three rules: first the particles must be more fluorescent than the background (this can be ensured by increasing the threshold until only the particles remain). Second the particle must have a clear and circular shape. Finally, the particle must be located on the aneurysm inner wall and not in the cavity or outside the defined area (figure S7.e). The sum of particles in all the slices located in the aneurysm was then divided by the total surface area of the slices (product of the sum of circumferences and the thickness) to obtain concertation in the aneurysm. The same of then done for the control vessels.

The results shown in figure S7.f reveal that two aneurysms showed similar results, but one had a significantly more particles in the aneurysm as well as in the control artery. However, since the LI was similar in all cases (figure 4.d), a possible explanation would be a higher flow rate that resulted in a higher particle flux. As we have seen from the in-vitro results, GPVI particles are able to adhere in a wide range of physiological WSS. Thus, an increase of shear within that range, possibly due to increase heart rate, will inevitably mean a proportional increase of deposited particle concentration throughout the vasculature.

Computational Fluid Dynamics

Equation solved as described in the Ansys Fluent manual [3] and the numerical setup and boundary condition for all the models used:

<u>Momentum:</u>

$$\frac{\partial}{\partial t}(\rho\vec{v}) + \nabla\cdot(\rho\vec{v}\,\vec{v}) = -\nabla p + \nabla\cdot(\bar{\bar{\tau}}) + \rho\vec{g} + \vec{F} \qquad (S1)$$

p - static pressure

$\rho\vec{g}$ and $\vec{F}$ - gravitational body force and external body forces, respectively.

$$\bar{\bar{\tau}} = \mu\left[(\nabla\vec{v} + \vec{v}^T) - \frac{2}{3}\nabla\cdot\vec{v}I\right] \qquad (S2)$$

μ - molecular viscosity

I - unit tensor,

$\frac{2}{3}\nabla\cdot\vec{v}I$ - volume dilation.

<u>Continuity:</u>

$$\frac{d\rho}{dt} + \nabla\cdot(\rho\vec{v}) = 0 \qquad (S3)$$

<u>Wall shear stress:</u>

$$\tau_w = \mu\frac{\partial\boldsymbol{u}}{\partial\boldsymbol{n}} \qquad (S4)$$

$\boldsymbol{u}$ - near wall velocity vector field.

$\boldsymbol{n}$ - wall normal vector.

Table S1 Numerical Setup

Model: Viscous Laminar<br>Outlet: zero pressure outlet<br>Wall: no slip<br>Inlet: Developed-Poiseuille 200 ml/min	Material:<br>Viscosity 3.5e-5 $Pa\cdot Sec$<br>Density: 1000 kg/m^3	Solver: coupled<br>Gradient: Least Square Cell Based<br>Pressure: Second Order<br>Momentum: Second order Upwind

Mesh Conversion

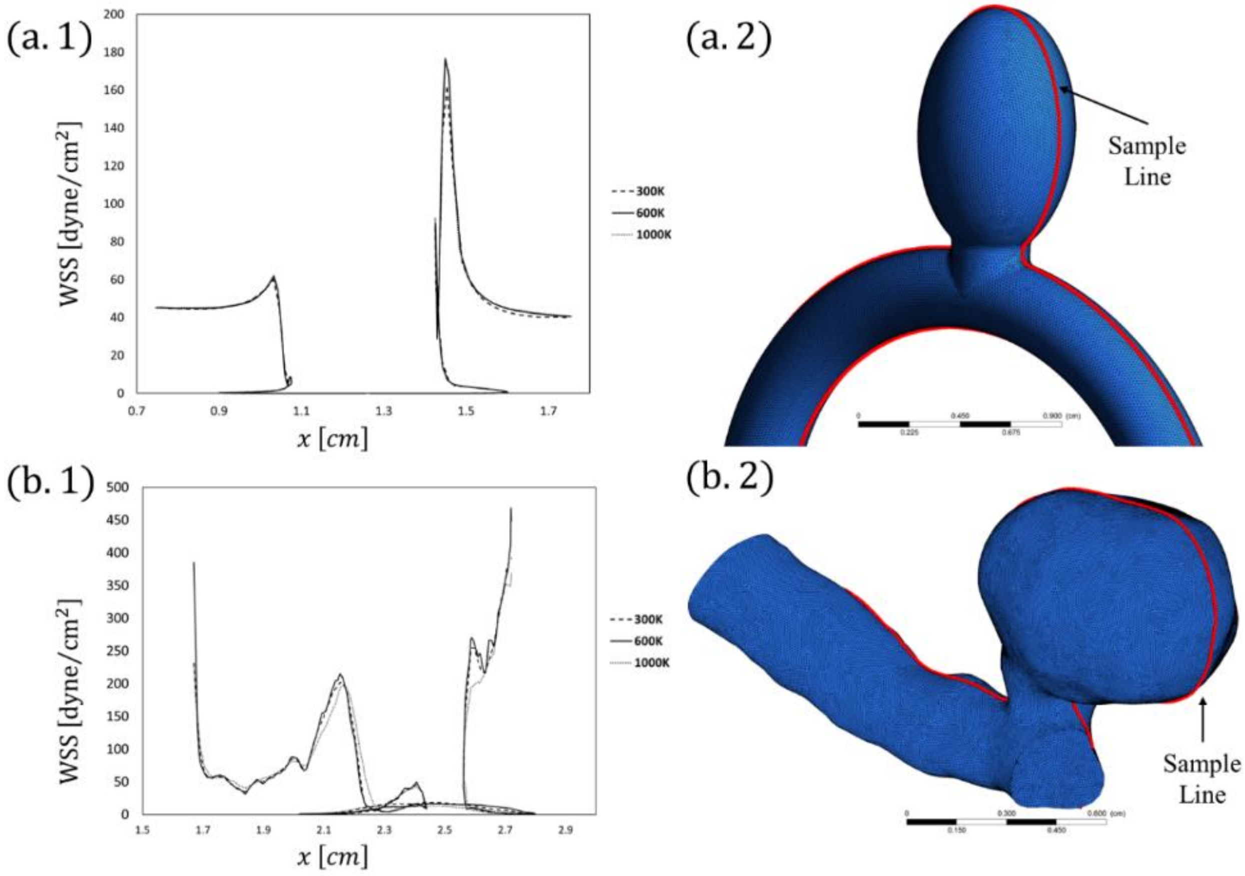

Figure S8 Mesh convergence studies (a.1) WSS vs position of the test line for the simlified model showing little difference between a 600 K and 1000K elements mesh particularly arount x=1.5 where the difference were more significan. (a.2) the final mesh containing 1000K tetrahedral elements with the sample line is marked in red. 1 (a.2) WSS vs position of the test line for thepatinet specific model showing little difference between a 600 K and 1000K elements mesh particularly arount x=2.7 where the difference were more significan. (a.2) the final mesh for the patient specific geometry containing 1000K tetrahedral elements with the sample line is marked in red.

Mesh convergence studies were done by doubling the number of elements for each mesh starting from 300K tetrahedral elements, bringing it to full convergence. The relative error of 10^{-9} was sufficient for the patient specific while 10^{-12} was used for the simplified model. Then, we plotted WSS on the red curve as shown in figure S8.b.2 and S8.a.2 for each mesh until no more significant change in the WSS distribution along the line was observed. For the simplified geometry we so little difference between 600k and 1000K elements (figure S8.a.1) and same was observed for the patient specific model (figure S8.b.1).

Particles

Bradford Assay Kit (Thermo scientific) was used to measure protein density.

Zeta potential was obtained via laser doppler micro-electrophoresis (Zetasizer Nano-ZS, Malvern Instruments, Malvern, UK).

Table S2 Summary of particle characteristics **NC: No Coating WC: With Coating**

Material	size	ζ potential	Coating	Ligand Density
PLGA	1750 ± 320 nm	-43.2 ± 9.18 mV	-	-
Polystyrene	246 ± 57 nm	-34.2 ± 5.7 mV	-	-
Polystyrene	1948 ± 103nm	-37.9 ± 11.7 mV	-	-
Polystyrene	1948 ± 103nm NC<br>4314 ± 126nm WC	-34.2 ± 5.7 mV NC<br>17.8 ± 8.2mV WC	GPVI	$4421.7 \pm 1813\ \#/\mu m^2$
Polystyrene	1647 ± 112nm NC<br>2743 ± 43nm WC	-34.4 ± 6.7 mV NC<br>-3.1 ± 6.5 mV	BSA	$10821.8 \pm 1263\#/\mu m^2$

Localization Index (LI)

To quantify the localization of the particles we first calculated the ratio of particle or fluorescence concertation (FC) for the cavity, the parent arteries or the control vessel (in the *in-vivo* model) which is the ratio of the total number of particles in a given region to the area of that region. Second, we defined the localization index as the ratio of FC for the cavity (FC_C) to the sum of FC in the cavity and the parent artery the aneurysm ($FC_C + FC_P$) as shown in equation S.5.

$$LI = FC_C/(FC_C + FC_P) \quad \text{(S.5)}$$

The meaning of LI is that if it equal 0.5 then there is no difference between the aneurysm in the parent artery while a value of 1 mean that all the particles localized to the aneurysm. Also, the ratio in equation S.6 is the ratio between the particles in the aneurysm and the parent or control artery.

$$A_P = LI/(1 - LI) \quad \text{(S.6)}$$

Particles Adhesion Kinetics

Forces on Particles

These forces depend on the relative acceleration of the particle toward the wall. All the particles in this study have very small relaxation time suggesting that most of them reach terminal velocity before hitting the wall in which case eq.1 can be used to calculate the forces.

$$F_d = F_g = (\rho_p - \rho_f) g \frac{3}{4} \pi R^3 = 0.05 \cdot 9.81 \cdot 0.75 \cdot \left(\frac{2}{10^6}\right) = \ 2.9 \cdot 10^{-18} N \qquad (eq. S1)$$

Where: ρ_p − particle density , ρ_p − fluid density ,g − gravity acceleration , R − Partilcle radius

In cases where particles are in various stage of acceleration F_g is constant and equal to $2.943 \cdot 10^{-18}$N but F_d changes and depends on the velocity and can be calculated from the Stokes drag formula (eq.2) if the velocity is known.

$$F_d = 6\pi R \mu v \qquad (eq. S2)$$

Where: μ − Fluid dynamic viscosty and v − Particle velocity

Targeting Ligand Specificity

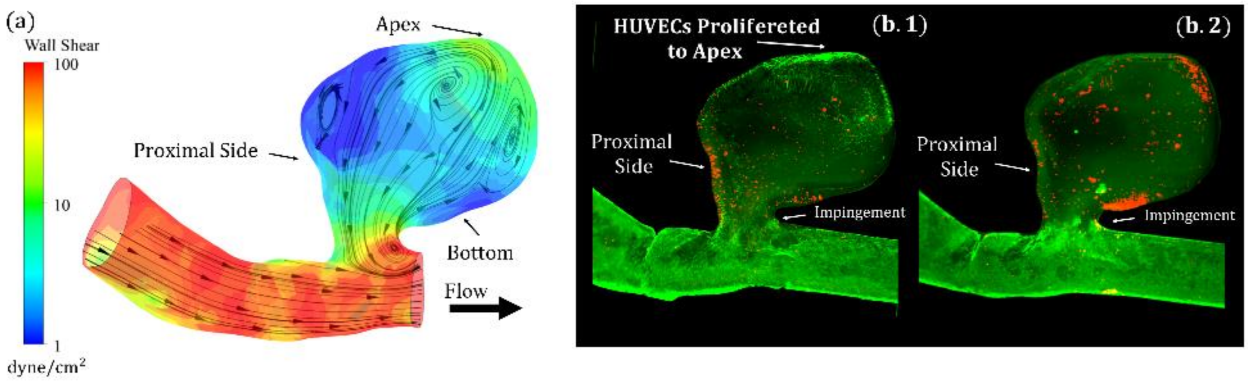

Figure S9 ***In-vitro*** **patient specific model characteristics** (a) CFD results on the patient specific Geometry showing a wide range of WSS ranging from less than 1 to 15 $dyne/cm^2$ within the aneurysm cavity .The results also show surface streamlines on the central plane revealing an medium shear zone at the apex (b.1) One of the patient specific injury models in which cells proliferated to the apex showing no adhesion of particles there but an enhanced deposition at the proximal side (b.2) One of the patient specific injury models in which there are no cells at the apex showing a lot of particles the but also little adhesion of at the proximal side.

The presence of some endothelial cells that proliferated to the apex changed somewhat the deposition pattern in the cavity (figure S9.b.1 vs S9.b.2). By deflecting GPVI coated particles away from the apex the cells allowed the particles to accumulate at proximal side (figure S9.b.1). However, in models with exposed collagen at the apex the particles adhered to the wall and were removed from circulation thus leaving the proximal side relatively free from particles (figure S9.b.2). This effect, highlight the process of target masking which we recently demonstrated [4] as well as some evidence the particles actually adhere to collagen and not endothelial cells.

Another, key aspect in the patient specific model is the presence of an impingement point at the distal side of the aneurysm neck on which particles impact and adhere regardless of surface characteristic or particle affinity to it. This fact is further illustrated by the adhesion of BSA coated particles on the distal neck of a fully endothelialized model which otherwise should have deflected

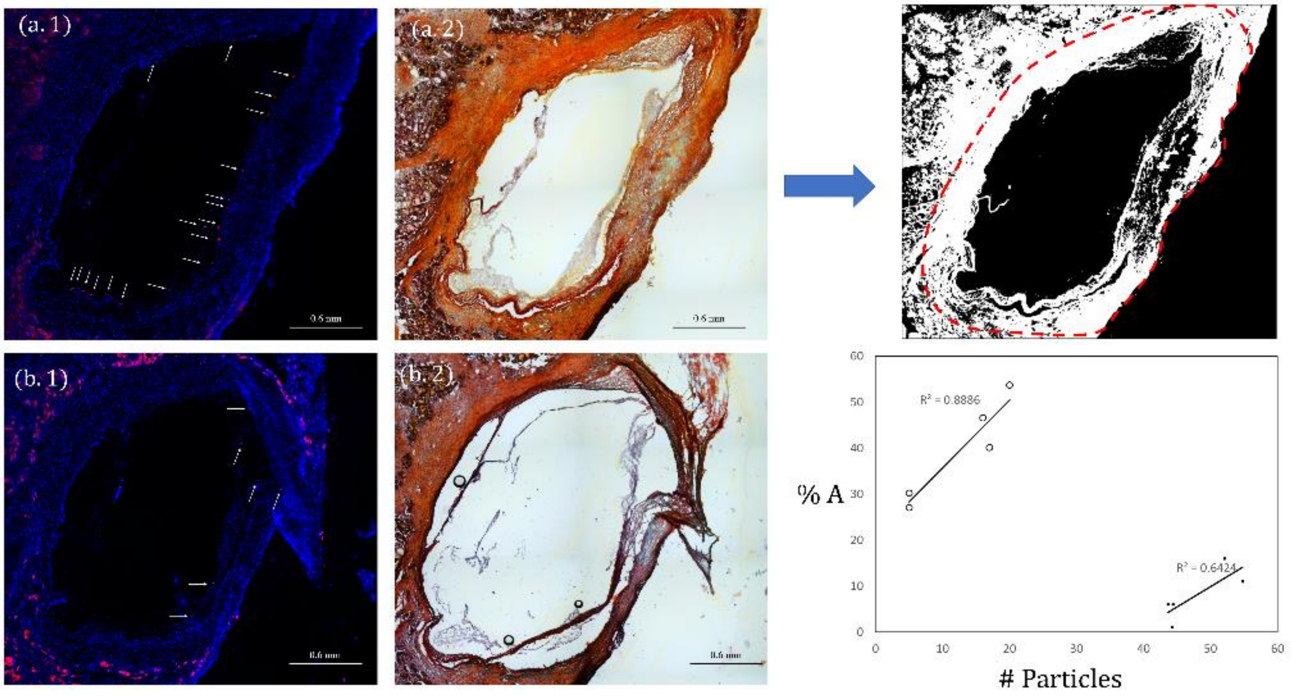

Figure S10 (a.1) 45μm thick, DAPI (4′,6-diamidino-2-phenylindole) stained slice taken from a rabbit aneurysm, 18 red fluorescent particles adhered to the inner surface of the aneurysm and marked by white arrows (a.2) Movats' pentachromic staining of the adjacent slice showing that the aneurysm wall is almost devoid of endothelial cells with exposed collagen and fibrin to which the particle (b.1) 45μm thick, DAPI stained slice taken from a rabbit aneurysm, 6 red fluorescent particles adhered to the inner surface of the aneurysm and marked by white arrows (b.2) Movats' pentachromic staining of the adjacent slice here showing less red and yellow color and more blue indicating less collagen and more mucin indicating greater presence of intact endothelial cells. (c) The image shows the marked area defining the aneurysm area according to criteria presented in figure S7 all the blue and green color were subtracted from the image and the remaining turned binary black and white, the percentage of the nonzero pixels within the defined area is considered the % of red within the aneurysm (%A) (d) Plot of %A vs number of particles in a slice showing good correlation in two aneurysms 1091 and 1088 with $R^2 = 0.886$ and $R^2 = 0.6474$ respectively.

them (see video SV4). All together this illustrates the variety of physiologically relevant conditions present in our in-vitro patient specific models.

A similar trend appears in the histological analysis of our in-vivo results. It can be seen that that there are more particles in slices with more collagen in the same aneurysm (figure S.10 a vs b). A correlation of the percentage of "red" within the aneurysm area which represents collagens, fibrins and elastin, to the number of particles in the slice in the same aneurysm shows that the more collagen is present within a slice the more particles it tends to have further suggesting the particles adhere to more exposed connective tissue than anything else.

Legends for Movies

Video SV1: Time lapse movie of 2 μm fluorescent particles circulation inside an in vitro aneurysm model showing slow circulatory flow within the cavity. The field of view was illuminated by a laser sheet (LaVision) and images were taken at a frame rate of 30 frames/s. Flow rate was 200ml/min. Inset: Picture of a 3D Computer-Aided-Design model of the aneurysm and its parent artery.

Video SV2: Time lapse movie showing green fluorescent 2 μm (right) and 200 nm (left) carboxylated polystyrene particles flowing and depositing in a simplified aneurysm model revealing increased deposition of 2 μm particles in the cavity associated with low WSS while the 200 nm deposited more profoundly in the parent artery and the impingement zone associated with high WSS. The final result after a washing step is presented at the end of the video. The images were acquired every 10 second over 50 minutes during the deposition experiments.

Video SV3: A time lapse movie showing red fluorescent 2 μm polystyrene particles in the patient specific aneurysm model revealing the flow patterns and particle trajectories. The particles near the bottom and the proximal side of the cavity are flowing slow enough to observe individual particles which correspond to the ultra-low shear in the region, while a rapid flow-direction change is visible near the apex representing higher shear. On the right: image of the particle deposition at the end of the experiment (see Figure 3) The movie shows images acquired every 10 seconds for 50 minutes. Flow rate was 200ml/min.

Video SV4: A time lapse movie showing a top view of a patient specific aneurysm in vitro model where red fluorescent 2 μm polystyrene coated with BSA deposit only the on the distal neck of a fully endothelialized model. The movie shows images acquired every 10 seconds for 50 minutes. Flow rate was 200ml/min.

MATLAB Codes

```
clc
clear all

%LOAD THE IMAGES OF THE SIMULATION (SIM.tif) AND THE
EXPERIMENTAL RESULTS (EX1.tif, EX2.tif, EX3.tif). ENFORCE
UNIFORM SIZE AND AXIS.

figure (1)
B= imread('SIM.tif');
sz=size(B);
hs = imshow(B);
figb = gcf;
axs = figb.CurrentAxes;

figure (2)
A1= imresize(imread('EX1.tif'),sz);
h1=imshow(A1);
figa1 = gcf;
ax1 = figa1.CurrentAxes;

figure (3)
A2= imresize(imread('EX2.tif'),sz);
h2=imshow(A2);
figa1 = gcf;
ax2 = figa1.CurrentAxes;

figure (4)
A3= imresize(imread('EX3.tif'),sz);
h3=imshow(A3);
figa1 = gcf;
ax3 = figa1.CurrentAxes;

nn=100; % RECTANGLE SIZE
k=1;
```

```
% CREATE A MOVING RECTANGLE AND CLCULATE THE AVERAGE
FLOURESCENE IN THE RECTANGLE WHILE BLOCKING EVERYTHING
OUTSIDE OF THE RECTANGLE. STORE THE RESULTS IN TWO VECTORS
ONE FOR THE SIMULATION (z) AND ONE FOR THE EXPERIMENTAL
RESULTS AVERAGED OVER ALL THREE (m).

for x = [1:nn:sz(2)-nn]
   for y=[1:nn:sz(1)-nn]

    e1 = imrect(ax1,[x y nn nn]);
    BW1 = createMask(e1,h1);
    ROI1 = A1;
    ROI1(BW1 == 0) = 0;

    e2 = imrect(ax2,[x y nn nn]);
    BW2 = createMask(e2,h2);
    ROI2 = A2;
    ROI2(BW2 == 0) = 0;

    e3 = imrect(ax3,[x y nn nn]);
    BW3 = createMask(e3,h3);
    ROI3 = A3;
    ROI3(BW2 == 0) = 0;

    es = imrect(axs,[x y nn nn]);
    BWs = createMask(es,hs);
    ROIs = B;
    ROIs(BWs == 0) = 0;


m(k)=mean(mean(mean(ROI1))+mean(mean(ROI2))+mean(mean(ROI3)
));
    err(k)=std([mean(mean(ROI1)) mean(mean(ROI2))
mean(mean(ROI3))]);
   z(k)=mean(mean(ROIs));
   k=k+1;
    end

sprintf('%d',x)
end
```

References

1. (2012) AneuriskWeb project website.

2. Epshtein, M., and Korin, N. (2018) Mapping the Transport Kinetics of Molecules and Particles in Idealized Intracranial Side Aneurysms. *Sci. Rep.*, **8** (1), 8528.

3. (2011) Ansys fluent theory guide. *ANSYS Inc., USA*, **15317**, 724–746.

4. Epshtein, M., and Korin, N. (2020) Computational and experimental investigation of particulate matter deposition in cerebral side aneurysms. *J. R. Soc. Interface*, **17** (169), 20200510.A
